# Supplementary material for: Dual-initiating and living frustrated Lewis pairs: expeditious synthesis of biobased thermoplastic elastomers
Source: Nat Commun. 2021 Aug 12;12:4874. doi: 10.1038/s41467-021-25069-6 (PMC8360971; doi:10.1038/s41467-021-25069-6)
Supplement: Supplementary file 1 — Supplementary Information [file 41467_2021_25069_MOESM1_ESM.pdf]

Supplementary Information

Dual-Initiating and Living Frustrated Lewis Pairs: Expeditious Synthesis of Biobased Thermoplastic Elastomers

Yun Bai,[1] Huaiyu Wang,[1] Jianghua He,[1] Yuetao Zhang[1]* & Eugene Y.-X. Chen[2]

[1] State Key Laboratory of Supramolecular Structure and Materials, College of Chemistry, Jilin University, Changchun, Jilin, 130012, China. E-mail: ytzhang2009@jlu.edu.cn

[2] Department of Chemistry, Colorado State University, Fort Collins, Colorado 80523-1872, United States.

Table of Contents

Supplementary Methods 1 - Materials, reagents, and methods 3

Supplementary Methods 2 - Synthesis of bifunctional organophosphorus superbases 5

Supplementary Methods 3 - Kinetic studies of bisfunctional organophosphorus superbases with different bridges. 21

Supplementary Methods 4 - Stoichiometric NMR Reaction 21

Supplementary Methods 5 - Select polymerization results.. 25

Supplementary Methods 6 - The optical properties of biorenewable PMMBL-based TPEs.. 27

Supplementary Methods 7 - Plots of M_n and $Đ$ of PMMBL vs MMBL conversion 27

Supplementary Methods 8 - X-ray diffraction data 28

Supplementary References 34

Supplementary Methods 1 - Materials, reagents, and methods

All syntheses and manipulations of air- and moisture-sensitive materials were carried out in flamed Schlenk-type glassware on a dual-manifold Schlenk line, a high-vacuum, or an argon-filled glovebox. Toluene, benzene and THF were refluxed over sodium/potassium alloy distilled under nitrogen atmosphere; hexane and CH_2Cl_2 were refluxed over CaH_2 distilled under nitrogen atmosphere. All solvents were stored over molecular sieves 4 Å. Benzene-d_6 and CD_2Cl_2 was dried over molecular sieves 4 Å. NMR spectra were recorded on a Bruker Avance II 500 (500 MHz, ^{1}H; 125 MHz, ^{13}C; 471 MHz, ^{19}F; 202 MHz, ^{31}P) or Zhongke-Niujin AS400 (400 MHz, ^{1}H) instrument at room temperature (RT). Chemical shifts for ^{1}H and ^{13}C spectra were referenced to internal solvent resonances and are reported as parts per million relatives to $SiMe_4$, whereas ^{19}F NMR spectra were referenced to external $CFCl_3$. Air sensitive NMR samples were conducted in Teflon-valve sealed J. Young-type NMR tubes.

Methyl methacrylate (MMA) was purchased from J&K and purified through process that MMA was first degassed and dried over CaH_2 overnight, followed by vacuum distillation. Further purification of MMA involved titration with tri(*n*-octyl)aluminum (Strem Chemicals) to a yellow end point,[1] followed by distillation under reduced pressure. 2-Ethoxyethyl methacrylate (EEMA) was purchased from J&K and dried over CaH_2 overnight, followed by vacuum distillation. *γ*-Methyl-*α*-methylene-*γ*-butyrolactone (MMBL) and *α*-methylene-*γ*-butyrolactone (MBL) were purchased from TCI and dried over CaH_2 overnight, followed by vacuum distillation. All purified monomers were stored in brown bottles inside a glovebox freezer at -30 ºC. *n*-BuLi (1.6 M solution in hexanes), phosphorus trichloride, 3-hydroxy-2-butanone, 1,3-diisopropylthiourea, azidotrimethylsilane, 1,2-bis(diphenylphosphinoethane), 1,2-bis(diphenylphosphinopropane), 1,2-bis(diphenylphosphinobutane) and 1,2-bis(diphenylphosphinohexane) were purchased from Energy Chemical. Trimethylaluminum, triisobutylaluminium and 2,6-di-tert-butyl-4-methylphenol were purchased from J&K. Literature procedures were employed for the preparation of the following compounds:

N,N'-diisopropylimidazolin-2-imine (NI^iPr)H,[2-3] methyl bis(2,6-di-^{t}Bu-4-methylphenoxy)aluminum ($(BHT)_2AlMe$),[4] isobutyl bis(2,6-di-^{t}Bu-4-methylphenoxy) aluminum ($(BHT)_2Al^iBu$),[5] diisobutyl(2,6-di-^{t}Bu-4-methylphenoxy)aluminum ($(BHT)Al^iBu_2$)[5] and $Ph(Cl)PC_2H_4P(Cl)Ph$.[6]

General Polymerization Procedures. Polymerizations were performed in 20 mL glass reactors inside the glovebox for ambient temperature (ca. 25 ºC) runs. In a typical polymerization procedure, a predetermined amount of a Lewis acid (LA) (4 equiv.), such as $(BHT)Al^iBu_2$·MMA, was first dissolved in 500 μL of MMA and toluene inside a glovebox. The polymerization was started by rapid addition of $\mu^{Et}[P(NI^iPr)Ph]_2$ solution (1 equiv.) via a gastight syringe to the above mixture under vigorous stirring. After the measured time interval, a 0.2 mL aliquot was taken from the reaction mixture via pipet and quickly quenched into a 4-mL vial containing 0.6 mL of undried "wet" $CDCl_3$ stabilized by 250 ppm of BHT-H; the quenched aliquots were later analyzed by ^1H NMR to obtain the percent monomer conversion data. After the polymerization was stirred for the stated reaction time then the reactor was taken out of the glovebox, and the reaction was quenched by addition of 5 mL of 5% HCl-acidified methanol. The quenched mixture was isolated by filtration and dried in a vacuum oven at 50 ºC to a constant weight.

Polymer Characterizations. Polymer number-average molecular weight (M_n) and molecular weight distributions ($Đ = M_w/M_n$) were measured by gel permeation chromatography (GPC) coupled with a Wyatt DAWAN 8+ light scattering (LS) detector at 35 ºC and a flow rate of 1 mL/min, with DMF (HPLC grade, containing 50 mmol/L LiBr) as an eluent on a Waters 1515 instrument equipped with Waters 4.6×30 mm guard column and three Waters WAT054466, WAT044226, WAT044223 columns (Polymer Laboratories: linear range of molecular weight = 500 - 4×10^6), or gel permeation chromatography (GPC) analyses were performed on a Waters 1515 instrument equipped with a guard column MIXED 7.5×50 mm PL column and two MIXED-C 7.5×300 columns and a differential refractive index detector using DMF (HPLC grade, containing 50 mmol/L LiBr) as the eluent at 35 ºC and a flow rate of 1 mL/min. The differential refractive index (DRI) increment (d*n*/d*c*) value of 0.0844 mL/g was used for

PMMBL and 0.0981 for PMBL. For PMMA, number-average molecular weight (M_n) and $Đ$ = M_w/M_n were measured by the GPC instrument calibrated with 10 PMMA standards, and chromatograms were processed with Waters Breeze 2 software.

Tensile Testing. TPEs films (thickness ~ 0.15 – 0.3 mm) were prepared by dissolving triblock copolymers in chloroform and then casting on glass plate. The solvent was then allowed to evaporate for 24 h at RT, and residual solvent was removed by drying vacuum for 24 h. Dog-bone-shaped specimens were die-cut from the prepared films with a width of 2.0 mm and a length of 12 mm. Uniaxial tensile tests were conducted on an Instron universal testing machine (Model 5944) equipped with a 10 N load cell operated at a crosshead speed of 10 mm/min. Tests were performed at least three measurements for each sample.

Transmittance measurement. Triblock copolymers were dissolving in chloroform and then casting on optical glass plate. The solvent was then allowed to evaporate for 24 h at RT, and residual solvent was removed by drying vacuum for 24 h. The transmittance was tested on a PerkinElmer LAMBDA 365 UV/Vis Spectrophotometer with a wavelength range of 200-800 nm and a scanning speed of 20 nm/second taking optical glass as reference.

Supplementary Methods 2 - Synthesis of bifunctional organophosphorus superbases

Supplementary Methods 2.1 - μ^{Et}[P(NI^{i}Pr)Ph]$_2$

***N*, *N*-(ethane-1,2-diylbis(phenylphosphanediyl))bis(1,3-diisopropyl-4,5-dimethyl-1,3-dihydro-2H-imidazol-2-imine) (μ^{Et}[P(NI^{i}Pr)Ph]$_2$).** It was prepared in the similar procedure as described for the synthesis of P(NI^{i}Pr)Ph_2.[7] In an argon-filled glovebox, a 200 mL Schlenk flask was equipped with a stir bar and charged with THF (80 mL) and (NI^{i}Pr)H (1.95 g, 10 mmol). This flask was sealed with a rubber septum, removed from the glovebox, interfaced to a Schlenk line, and then brought to -78 °C, where a solution of *n*-BuLi (1.6 M in hexane, 6.25 mL, 10 mmol) was added dropwise via syringe to the above flask. After completion of the addition, the cold bath was removed and the mixture was allowed to warm to RT and stirred at

RT for 3 h. It was cooled down to -78 °C again and added with Ph(Cl)PC_2H_4P(Cl)Ph[6] (1.58 g, 5 mmol, in 20 mL THF), then warmed to RT and stirred overnight. The volatiles were removed in vacuo and the residue (50:50 mix of *rac* and *meso* diastereomers) was dissolved in 90 mL of hexane. After filtration, the filtrate was collected and concentrated to 20 mL, then stored in the freezer, $\mu^{Et}[P(NI^{i}Pr)Ph]_2$ was obtained as white solid (about 1:8 mix of *rac* and *meso* diastereomers). Yield 45% (1.43 g, 2.26 mmol). ^{1}H NMR (500 MHz, Benzene-d_6) δ 7.92 - 7.88 (m, 4H, Ph), 7.32 - 7.26 (m, 4H, Ph), 7.16 - 7.12 (m, 2H, Ph), 5.11 (s, br, 4H, NC*H*Me$_2$), 2.53 - 2.44 (m, 2H, PCH_2), 2.38 - 2.31 (m, 2H, PCH_2), 1.65 (s, 12H, 2CH_3), 1.26 (d, J = 7 Hz, 12H, NCHCH_3), 1.12 ppm (d, J = 7 Hz, 12H, NCHCH_3). ^{31}P NMR (202 MHz, Benzene-d_6) δ 42.2 (m) and 41.7 (m) ppm. ^{13}C NMR (125 MHz, Benzene-d_6) δ 150.0, 130.1, 127.0, 115.4, 46.2, 34.2, 21.6, 10.0 ppm.

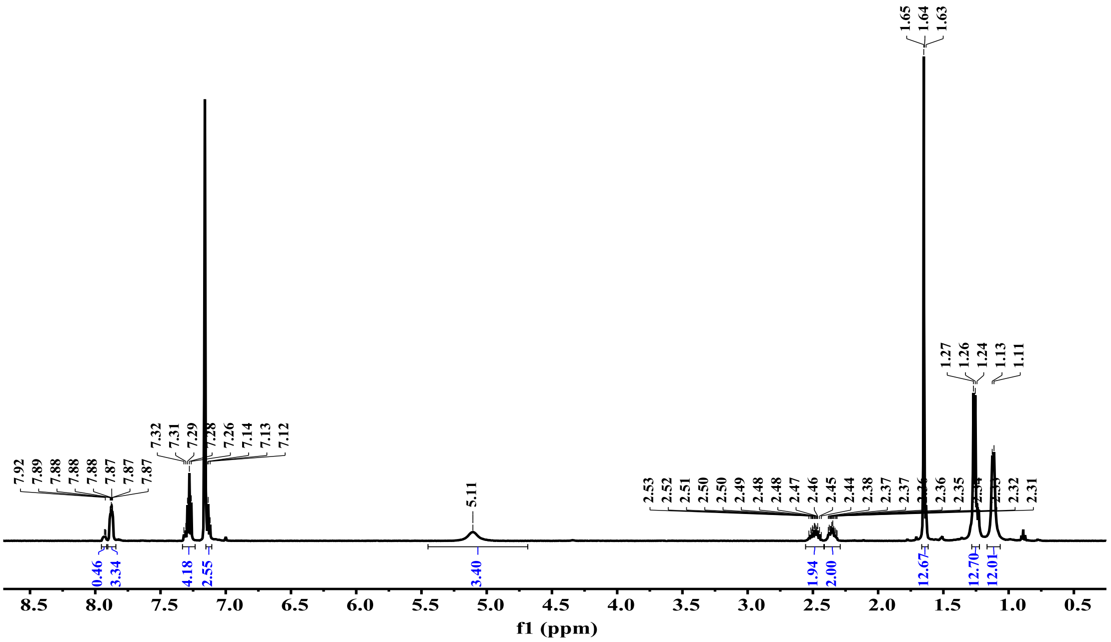

Supplementary Fig.1. ^{1}H NMR spectrum of $\mu^{Et}[P(NI^{i}Pr)Ph]_2$ (Benzene-d_6, 500 MHz).

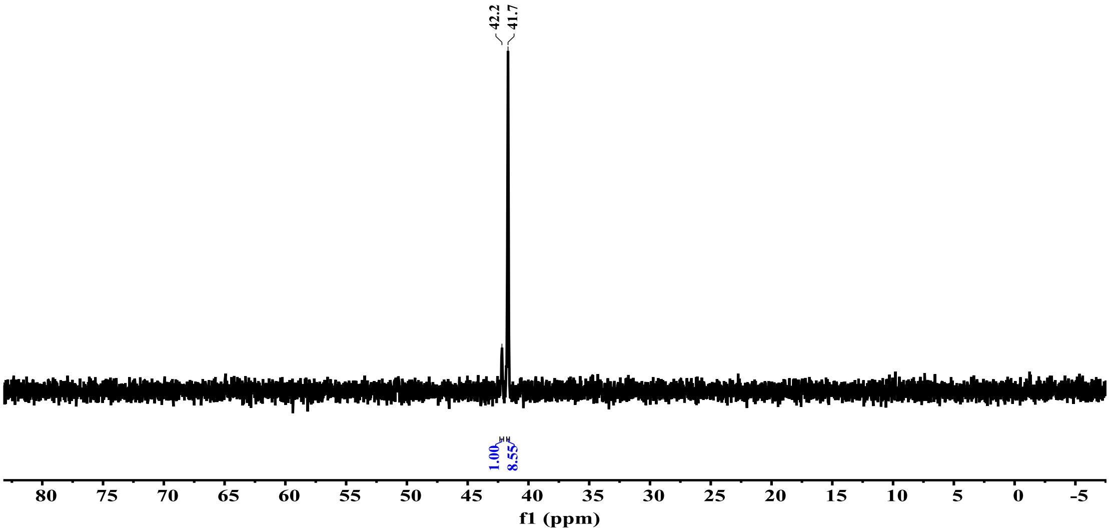

Supplementary Fig. 2. ^{31}P NMR spectrum of μ^{Et}[P(NI^{i}Pr)Ph]$_2$ (Benzene-d_6, 202 MHz).

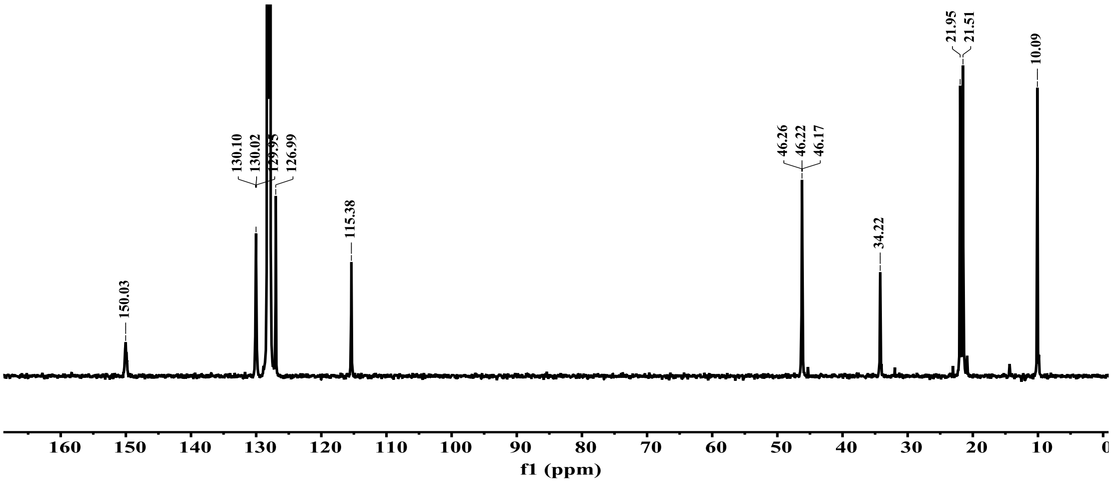

Supplementary Fig. 3. ^{13}C NMR spectrum of μ^{Et}[P(NI^{i}Pr)Ph]$_2$ (Benzene-d_6, 125 MHz).

Supplementary Methods 2.2 - μ^{Pr}[P(NI^{i}Pr)Ph]$_2$

[PhPC$_3$H$_6$PPh]$^{2-}$Li$^{+}_{2}$(THF)$_4$. [PhPC$_3$H$_6$PPh]$^{2-}$Li$^{+}_{2}$(THF)$_4$ was prepared in the similar procedure as described from literature.[6] A solution of 1,2-bis(diphenylphosphinopropane) (10.3 g, 25 mmol) in THF (100 mL) was added slowly to a rapidly stirred THF suspension of Li (1.75 g, 250 mmol) at 0 °C. The mixture was allowed to warm to RT and stirred for 24 h. The mixture was decanted from the excess Li and the solvent reduced by half and cooled to -15 °C. The yellow precipitate was collected by filtration, washed with cold THF (30 mL) and hexane (2 × 25 mL), and then dried under vacuum (10.9 g, 19.5 mmol, 78% yield). ^{1}H NMR (500 MHz, Benzene-d_6): δ 7.54 (d, J = 7.5 Hz, 4H, Ar-H), 7.16 (m, 4H, Ar-H), 6.80 (t, J = 7.5

Hz, 2H, Ar-*H*), 3.46 (m, 16H, OCH_2CH_2), 2.68 (br, 4H, PCH_2), 2.54 (br, 2H, PCH_2), 1.31 (m, 16H, OCH_2CH_2). ^{31}P NMR (202 MHz, Benzene-d_6) δ -70.9 (br) and 79.1 (br) ppm.

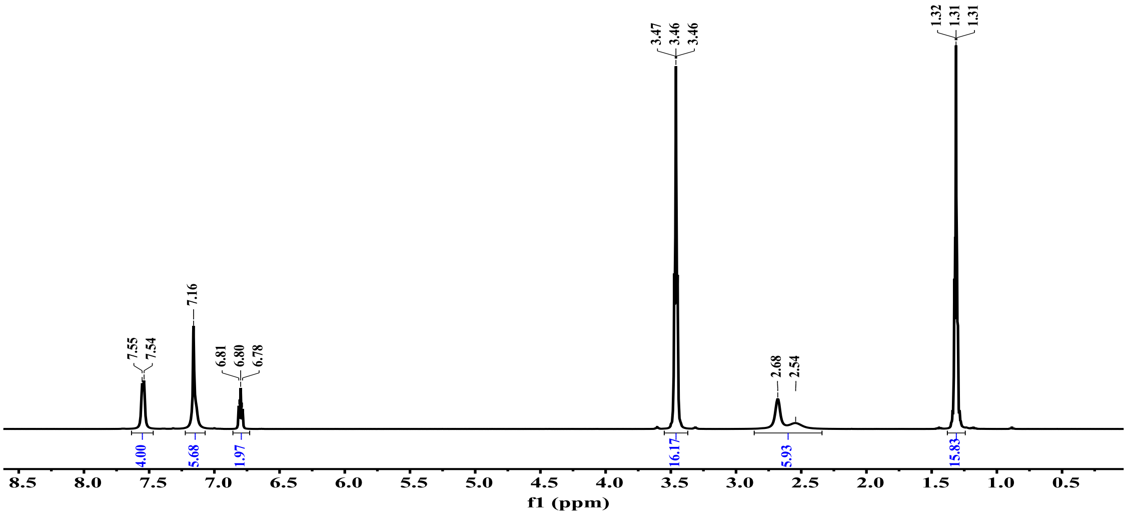

Supplementary Fig.4. ^{1}H NMR spectrum of $[PhPC_3H_6PPh]^{2-}Li^+_2(THF)_4$ (Benzene-d_6, 500 MHz).

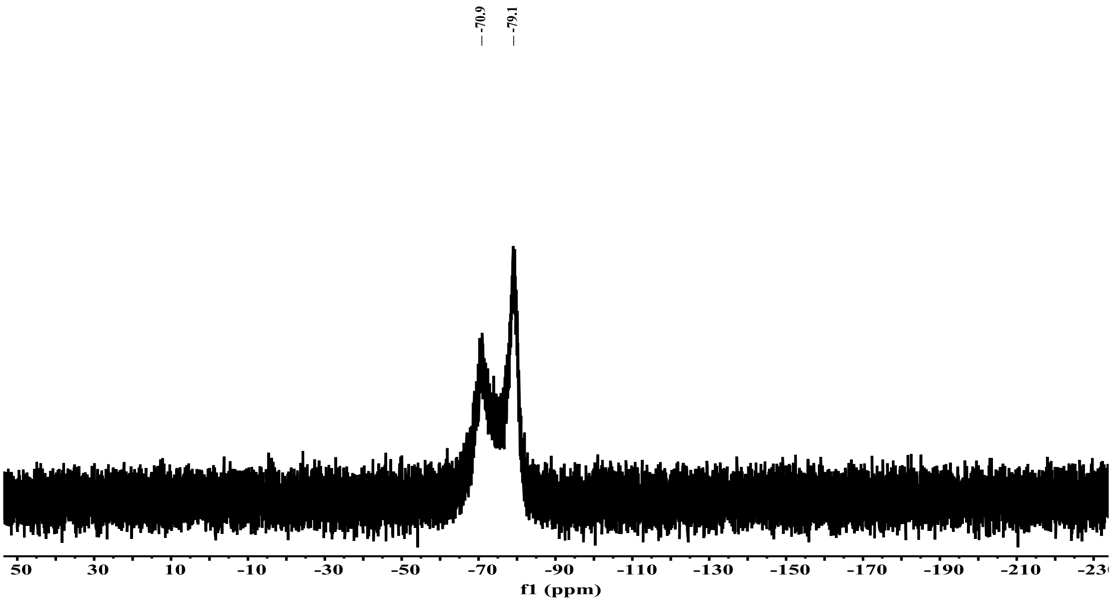

Supplementary Fig.5. ^{31}P NMR spectrum of $[PhPC_3H_6PPh]^{2-}Li^+_2(THF)_4$ (Benzene-d_6, 202 MHz).

Ph(Cl)PC$_3$H$_6$P(Cl)Ph. An ether (40 mL) solution of $[PhPC_3H_6PPh]^{2-}Li^+_2(THF)_4$ (3.72 g, 6.64 mmol) cooled at -78 °C was slowly added with PCl_3 (2.9 mL, 33.2 mmol). The mixture was stirred for 30 min at -78 °C and 30 min at 25 °C. After filtration, the volatiles of filtrate were removed in vacuo, furnishing colorless oil product (1.82 g, 5.5 mmol, 83% yield). ^{1}H NMR

(500 MHz, Benzene-d_6): δ 7.50-7.47 (m, 4H, Ar-*H*), 7.05-7.03 (m, 6H, Ar-*H*), 1.95-1.90 (m, 2H, PC*H*$_2$), 1.69-1.58 (m, 4H, PC*H*$_2$) ppm. ^{31}P NMR (202 MHz, Benzene-d_6): δ 91.0 (m) ppm. ^{13}C NMR (125 MHz, Benzene-d_6): δ 138.8, 131.1, 128.8, 38.1, 20.2 ppm.

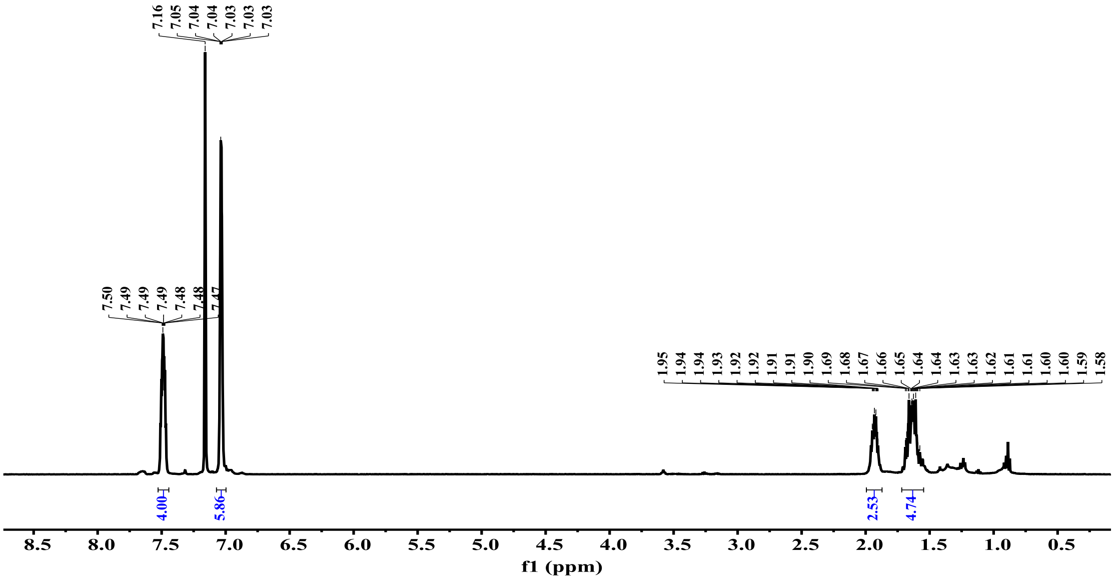

Supplementary Fig.6. ^{1}H NMR spectrum of Ph(Cl)PC$_3$H$_6$P(Cl)Ph (Benzene-d_6, 500 MHz).

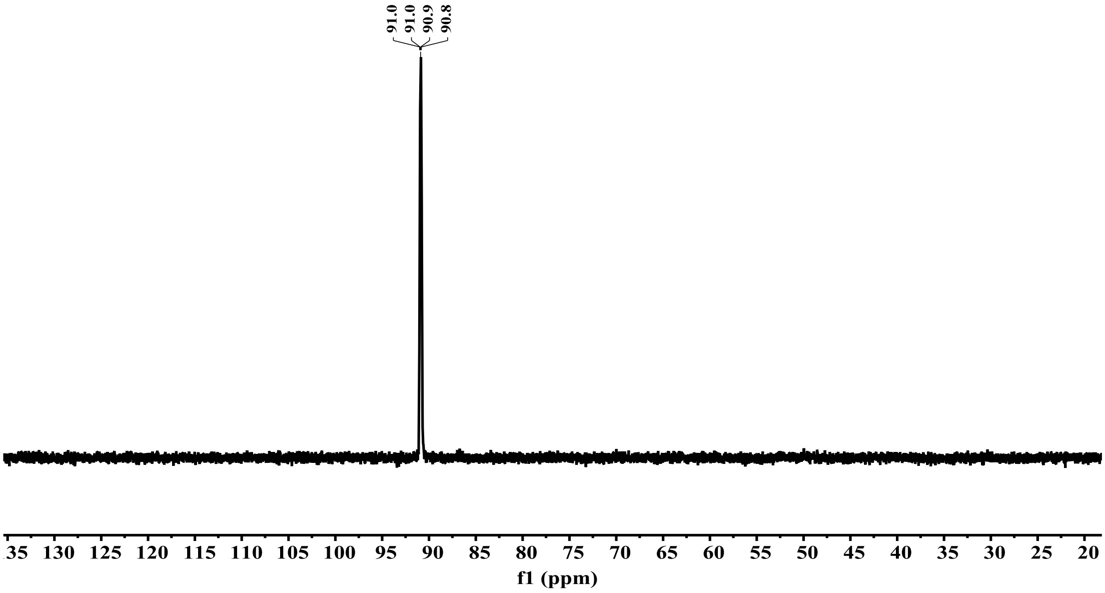

Supplementary Fig.7. ^{31}P NMR spectrum of Ph(Cl)PC$_3$H$_6$P(Cl)Ph (Benzene-d_6, 202 MHz).

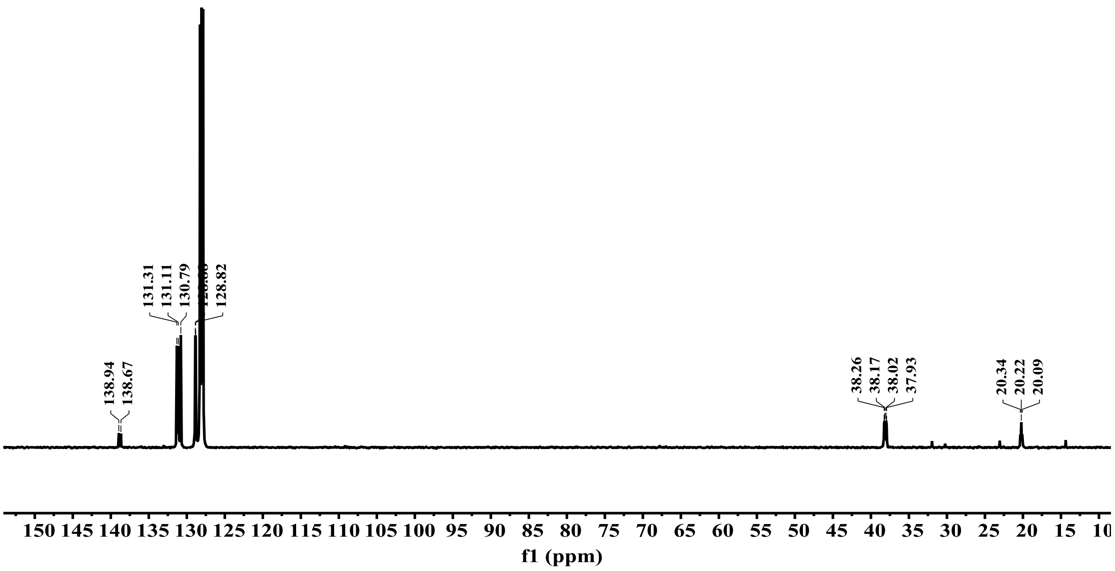

Supplementary Fig.8. ^{13}C NMR spectrum of Ph(Cl)PC_3H_6P(Cl)Ph (Benzene-d_6, 125 MHz).

N, N'-(propane-1,3-diylbis(phenylphosphanediyl)) bis(1,3-diisopropyl-4,5- dimethyl-1,3-dihydro-2H-imidazol-2-imine) (μ^{Pr}[P(NI^{i}Pr)Ph]$_2$). It was prepared and isolated as white solid in 71% yield using the same procedure as described for the preparation of μ^{Et}[P(NI^{i}Pr)Ph]$_2$. ^{1}H NMR (500 MHz, Benzene-d_6) δ 7.91 - 7.88 (m, 4H, Ph), 7.38 - 7.28 (m, 4H, Ph), 7.16 - 7.14 (m, 2H, Ph), 5.10 (s, br, 4H, NC*H*Me$_2$), 2.34 - 2.30 (m, 4H, PC*H*$_2$), 2.20 - 2.17 (m, 2H, PC*H*$_2$), 1.65 (s, 12H, 2C*H*$_3$), 1.26 (d, J = 7 Hz, 12H, NCHC*H*$_3$), 1.11 ppm (d, J = 7 Hz, 12H, NCHC*H*$_3$). ^{31}P NMR (202 MHz, Benzene-d_6) δ 38.5(m) ppm. ^{13}C NMR (125 MHz, Benzene-d_6) δ 150.3, 130.0, 127.0, 115.4, 46.2, 42.1, 21.5, 10.0 ppm.

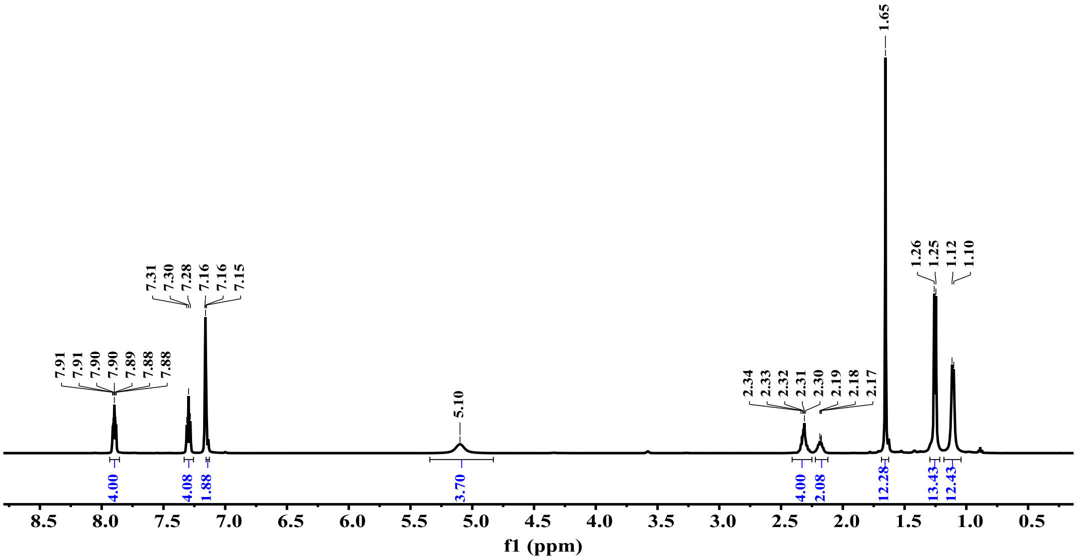

Supplementary Fig.9. ^{1}H NMR spectrum of μ^{Pr}[P(NI^{i}Pr)Ph]$_2$ (Benzene-d_6, 500 MHz).

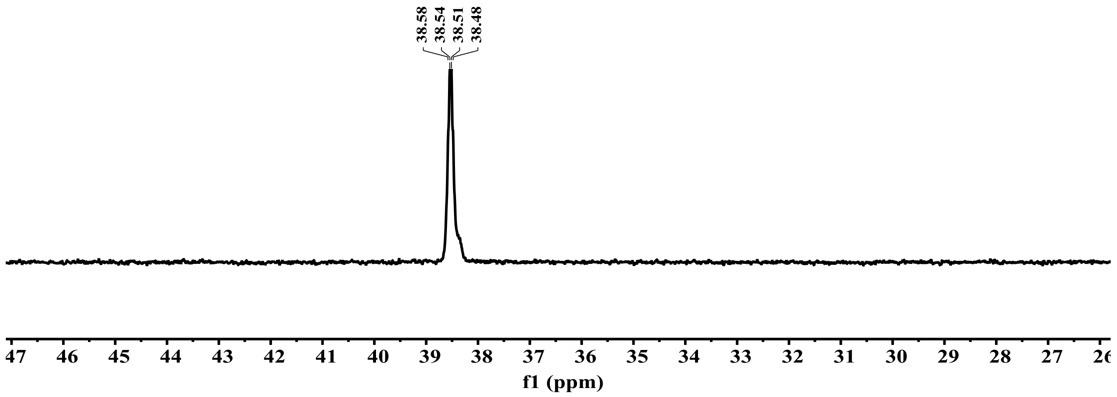

Supplementary Fig.10. ^{31}P NMR spectrum of μ^{Pr}[P(NI^{i}Pr)Ph]$_2$ (Benzene-d_6, 202 MHz).

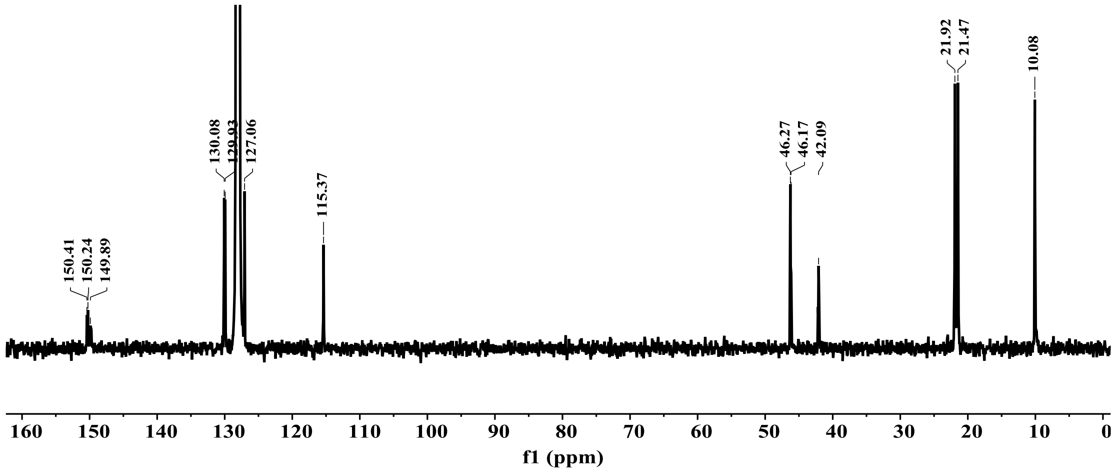

Supplementary Fig.11. ^{13}C NMR spectrum of μ^{Pr}[P(NI^{i}Pr)Ph]$_2$ (Benzene-d_6, 125 MHz).

Supplementary Methods 2.3 - μ^{Bu}[P(NI^{i}Pr)Ph]$_2$

[PhPC$_4$H$_8$PPh]$^{2-}$Li$^+_2$(THF)$_4$. A solution of 1,2-bis(diphenylphosphinobutane) (10.7 g, 25 mmol) in THF (100 mL) was added slowly to a rapidly stirred THF suspension of Li (1.75 g, 250 mmol). The mixture was allowed to warm to RT and stirred for 12 h. The mixture was decanted from the excess Li and the solvent was reduced by half and cooled to -15 °C. The yellow precipitate was collected by filtration, washed with cold THF (30 mL) and pentane (2 × 25 mL), and then dried under vacuum (10.37 g, 18.9 mmol, 76% yield).^{1}H NMR (500 MHz, Benzene-d_6): δ 7.55 (m, 4H, Ar-H), 7.15 (m, 4H, Ar-H), 6.80 (t, J = 8.0 Hz, 2H, Ar-H), 3.48 (m, 16H, OCH_2CH$_2$), 2.59 (br, 4H, PCH_2), 2.39 (br, 4H, PCH_2), 1.31 (m, 16H, OCH$_2$CH_2). ^{31}P NMR (202 MHz, Benzene-d_6) δ -72.7 (m) ppm.

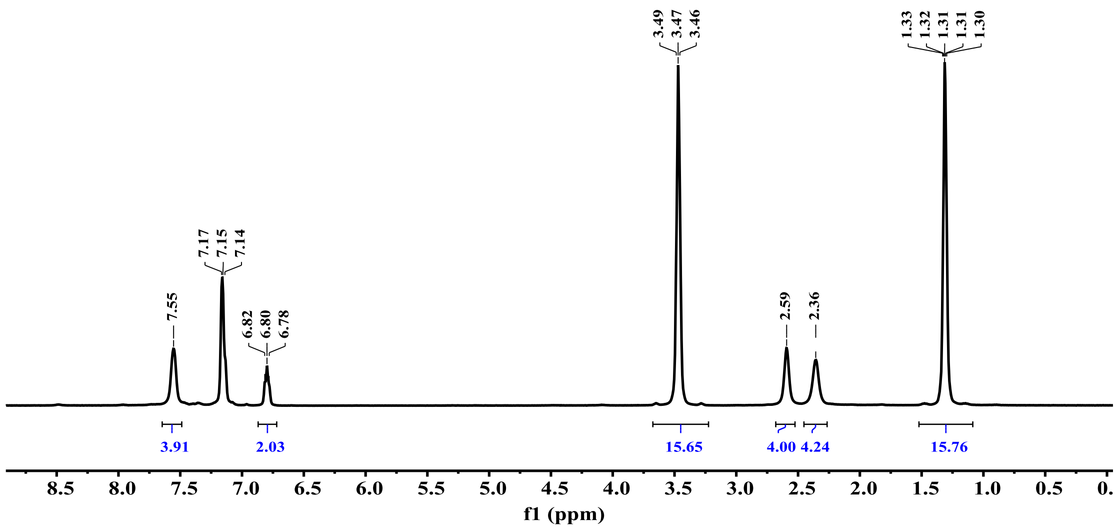

Supplementary Fig.12. ^{1}H NMR spectrum of $[PhPC_4H_8PPh]^{2-}Li^{+}_2(THF)_4$ (Benzene-d_6, 400 MHz).

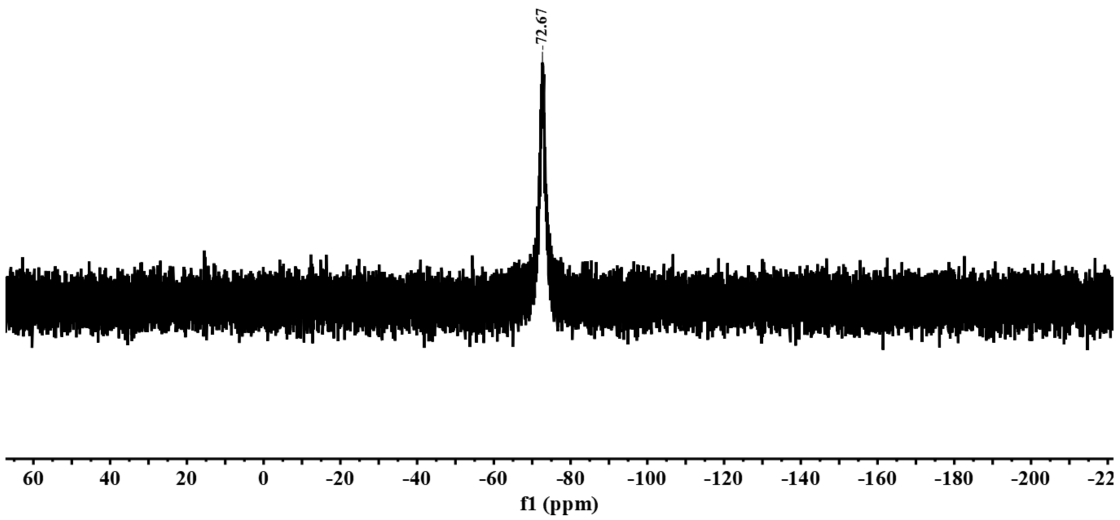

Supplementary Fig.13. ^{31}P NMR spectrum of $[PhPC_6H_{12}PPh]^{2-}Li^{+}_2(THF)_4$ (Benzene-d_6, 202 MHz).

Ph(Cl)PC$_4$H$_8$P(Cl)Ph. An ether (40 mL) solution of $[PhPC_4H_8PPh]^{2-}Li^{+}_2(THF)_4$ (3.8 g, 6.64 mmol) cooled to -78 ºC was slowly added with PCl_3 (2.9 mL, 33.2 mmol). The mixture was stirred for 30 min at -78 ºC and 30 min at 25 ºC. After filtration, the volatiles of filtrate were removed in vacuo, furnishing white solid product (2.1 g, 5.7 mmol, 87% yield). ^{1}H NMR (500 MHz, Benzene-d_6): δ 7.53-7.51 (m, 4H, Ar-H), 7.08-7.06 (m, 6H, Ar-H), 1.84-1.77 (m, 2H, PCH_2), 1.56-1.48 (m, 2H, PCH_2) 1.37-1.25 (m, 4H, PCH_2) ppm. ^{31}P NMR (202 MHz, Benzene-d_6): δ 92.7 (m) ppm. ^{13}C NMR (125 MHz, Benzene-d_6): δ 138.8, 131.3, 130.7, 128.8,

36.8, 25.9 ppm.

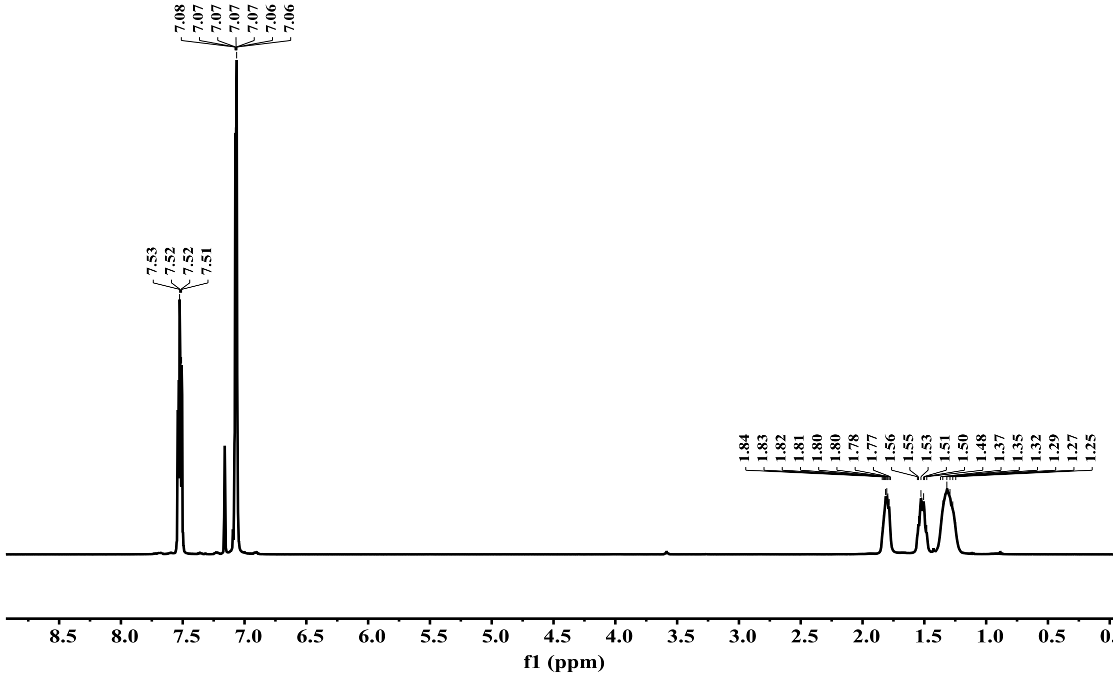

Supplementary Fig.14. ^{1}H NMR spectrum of $Ph(Cl)PC_4H_8P(Cl)Ph$ (Benzene-d_6, 500 MHz).

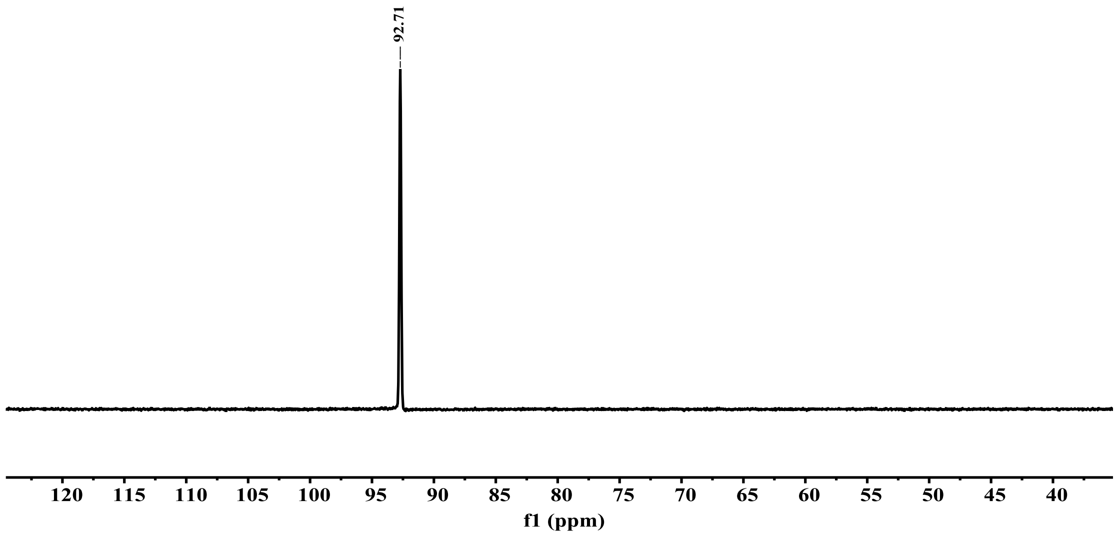

Supplementary Fig.15. ^{31}P NMR spectrum of $Ph(Cl)PC_4H_8P(Cl)Ph$ (Benzene-d_6, 202 MHz).

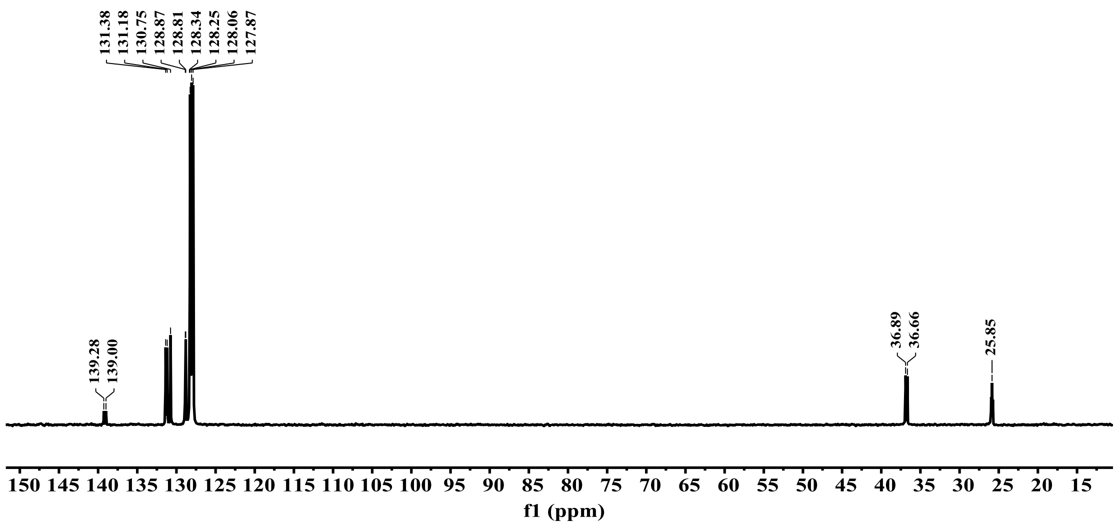

Supplementary Fig.16. [13] C NMR spectrum of $Ph(Cl)PC_4H_8P(Cl)Ph$ (Benzene-d_6, 125 MHz).

N, N'-(butane-1,6-diylbis(phenylphosphanediyl)) bis(1,3-diisopropyl-4,5-dimethyl-1,3-dihydro-2H-imidazol-2-imine) (μ^{Bu}[P(NI^{i}Pr)Ph]$_2$). It was prepared and isolated as white solid in 79% yield using the same procedure as described for the preparation of μ^{Et}[P(NI^{i}Pr)Ph]$_2$. ^{1}H NMR (500 MHz, Benzene-d_6) δ 7.88 - 7.85 (m, 4H, Ph), 7.31 - 7.27 (m, 4H, Ph), 7.16 - 7.13 (m, 2H, Ph), 5.08 (s, br, 4H, NC*H*Me$_2$), 2.17 - 2.11 (m, 4H, PC*H*$_2$), 2.08 – 1.96 (m, 4H, PC*H*$_2$), 1.66 (s, 12H, 2C*H*$_3$), 1.25 (d, *J* = 7 Hz, 12H, NCHC*H*$_3$), 1.11 ppm (d, *J* = 7 Hz, 12H, NCHC*H*$_3$). ^{31}P NMR (202 MHz, Benzene-d_6) δ 38.5(m) ppm. ^{13}C NMR (125 MHz, Benzene-d_6) δ 150.2, 149.9, 129.9, 127.1, 115.4, 46.2, 40.0, 27.7, 21.7, 10.1 ppm.

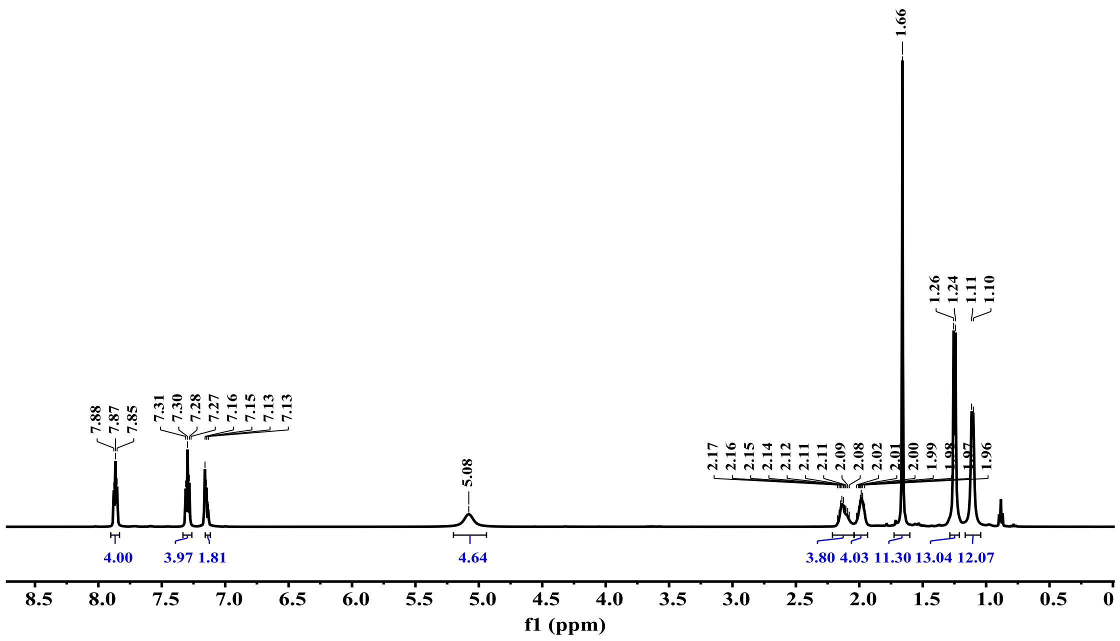

Supplementary Fig.17. ^{1}H NMR spectrum of μ^{Bu}[P(NI^{i}Pr)Ph]$_2$ (Benzene-d_6, 500 MHz).

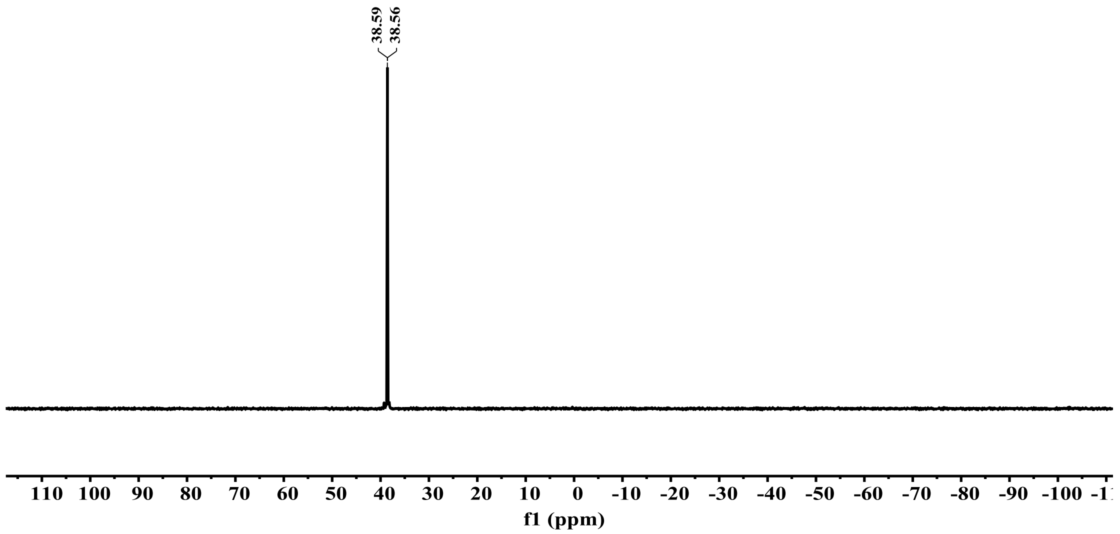

Supplementary Fig.18. ^{31}P NMR spectrum of μ^{Bu}[P(NI^{i}Pr)Ph]$_2$ (Benzene-d_6, 202 MHz).

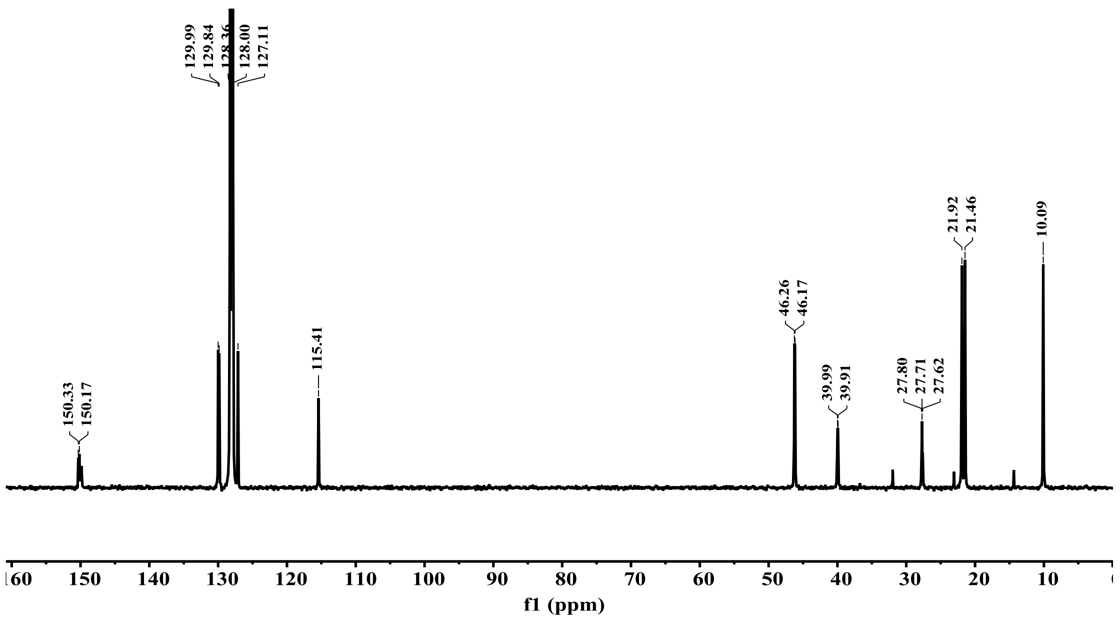

Supplementary Fig.19. ^{13}C NMR spectrum of μ^{Bu}[P(NI^{i}Pr)Ph]$_2$ (Benzene-d_6, 125 MHz).

Supplementary Methods 2.4 - μ^{Hex}[P(NI^{i}Pr)Ph]$_2$

Synthesis of [PhPC$_6$H$_{12}$PPh]$^{2-}$Li$^{+}_2$(THF)$_4$. A solution of 1,2-bis(diphenylphosphinohexane) (11.7 g, 25 mmol) in THF (100 mL) was added slowly to a rapidly stirred THF suspension of Li (1.75 g, 250 mmol). The mixture was allowed to warm to RT and stirred for 12 h. The mixture was decanted from the excess Li and the solvent reduced by half and cooled to -15 ºC. The yellow precipitate was collected by filtration, washed with cold THF (30 mL) and pentane (2 × 25 mL), and then dried under vacuum (11.87 g, 19.3 mmol, 77% yield).^{1}H NMR (500 MHz, Benzene-d_6): δ 7.54 (m, 4H, Ar-*H*), 7.16 (m, 4H, Ar-*H*), 6.79 (t, J = 7.5 Hz, 2H, Ar-*H*), 3.51 (m, 16H, OCH_2CH$_2$), 2.48 (br, 4H, PCH_2), 2.09 (br, 4H, PCH_2), 1.97 (br, 2H, PCH_2), 1.34 (m, 16H, OCH$_2$CH_2). ^{31}P NMR (202 MHz, Benzene-d_6) δ -65.9 (m) ppm.

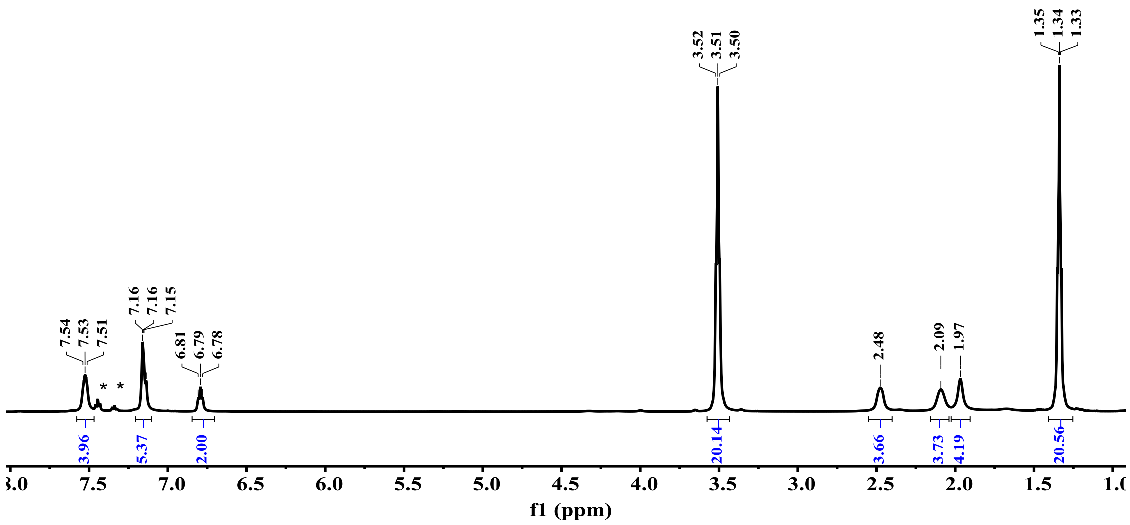

Supplementary Fig.20. ^{1}H NMR spectrum of $[PhPC_6H_{12}PPh]^{2-}Li^{+}_2(THF)_4$ (Benzene-d_6, 500 MHz) *was the unreacted 1,2-bis(diphenylphosphinohexane).

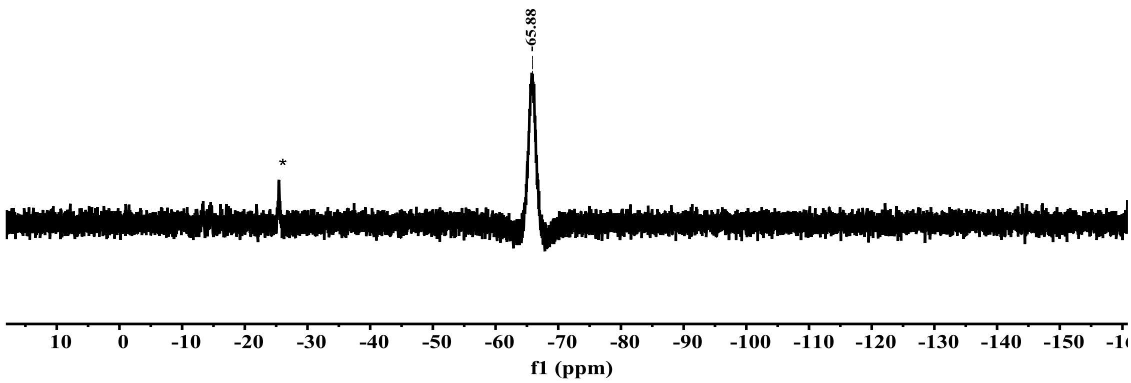

Supplementary Fig.21. ^{31}P NMR spectrum of $[PhPC_6H_{12}PPh]^{2-}Li^{+}_2(THF)_4$ (Benzene-d_6, 202 MHz) *was denoted for the unreacted 1,2-bis(diphenylphosphinohexane).

Synthesis of Ph(Cl)PC$_6$H$_{12}$P(Cl)Ph. To an ether (40 mL) solution of $[PhPC_6H_{12}PPh]^{2-}Li^{+}_2(THF)_4$ (4.0 g, 6.64 mmol) cooled to -78 °C was slowly added PCl_3 (2.9 mL, 33.2 mmol). The mixture was stirred for 30 min at -78 °C and 30 min at 25 °C. After filtration, the volatiles of filtrate were removed in vacuo, furnishing colorless oil product (2.16 g, .5.8 mmol, 88% yield). ^{1}H NMR (500 MHz, Benzene-d_6): δ 7.61-7.57 (m, 4H, Ar-*H*), 7.10-7.06 (m, 6H, Ar-*H*), 1.96-1.90 (m, 2H, PCH_2), 1.69-1.64 (m, 2H, PCH_2) 1.36-1.25 (m, 4H, PCH_2), 1.01-0.97 (m, 4H, PCH_2) ppm. ^{31}P NMR (202 MHz, Benzene-d_6): δ 93.1 (m) ppm. ^{13}C NMR (125 MHz, Benzene-d_6): δ 139.4, 131.2, 130.8, 128.8, 37.3, 30.3, 24.6 ppm.

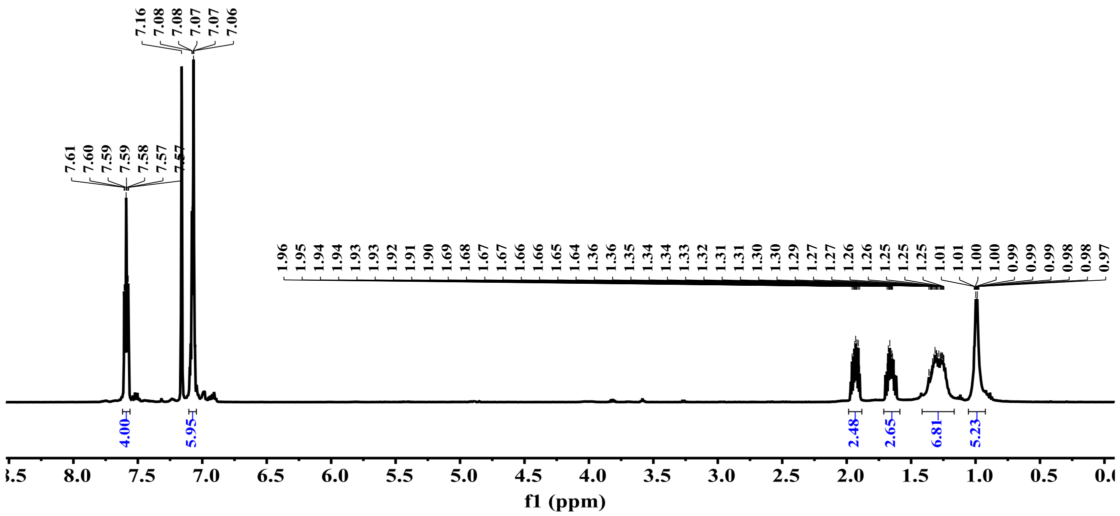

Supplementary Fig.22. ^{1}H NMR spectrum of Ph(Cl)PC_6H_{12}P(Cl)Ph (Benzene-d_6, 500 MHz).

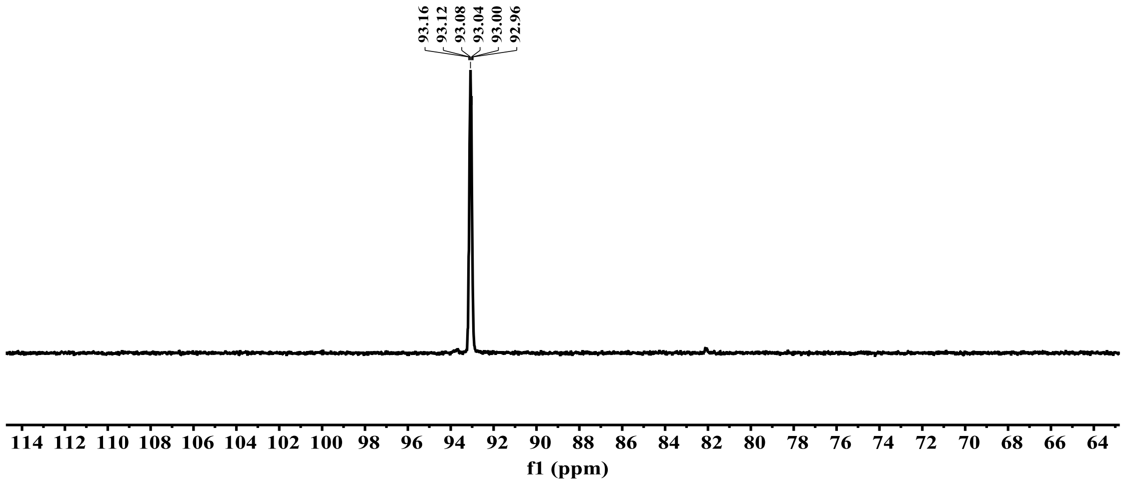

Supplementary Fig.23. ^{31}P NMR spectrum of Ph(Cl)PC_6H_{12}P(Cl)Ph (Benzene-d_6, 202 MHz).

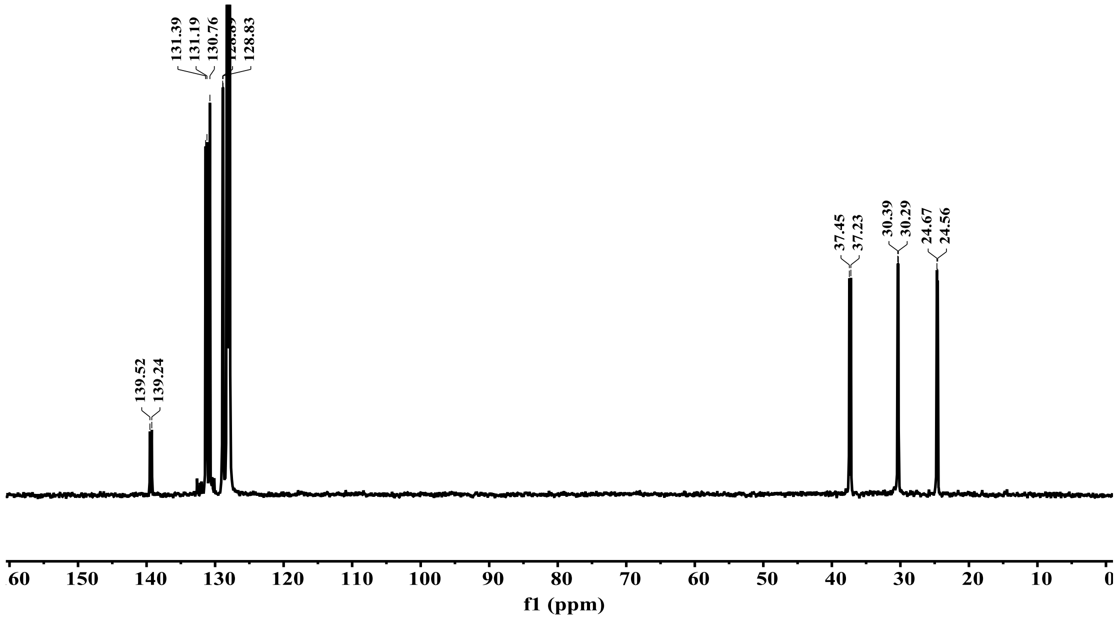

Supplementary Fig.24. ^{13}C NMR spectrum of Ph(Cl)PC_6H_{12}P(Cl)Ph (Benzene-d_6, 125 MHz).

Synthesis of N, N'-(hexane-1,6-diylbis(phenylphosphanediyl)) bis(1,3-diisopropyl-4,5 -dimethyl-1,3-dihydro-2H-imidazol-2-imine) (μ^{Hex}[P(NI^{i}Pr)Ph]$_2$). It was prepared and isolated as white solid in 74% yield using the same procedure as described for the preparation of μ^{Et}[P(NI^{i}Pr)Ph]$_2$. ^{1}H NMR (500 MHz, Benzene-d_6) δ 7.92 - 7.88 (m, 4H, Ph), 7.34 - 7.31 (m, 4H, Ph), 7.18 - 7.16 (m, 2H, Ph), 5.09 (s, br, 4H, NC*H*Me$_2$), 2.08 - 2.04 (m, 2H, PC*H*$_2$), 1.93 – 1.88 (m, 4H, PC*H*$_2$), 1.82 – 1.79 (m, 2H, PC*H*$_2$), 1.66 (s, 12H, 2C*H*$_3$), 1.59 – 1.56 (m, 4H, PC*H*$_2$), 1.26 (d, J = 7 Hz, 12H, NCHC*H*$_3$), 1.12 ppm (d, J = 7 Hz, 12H, NCHC*H*$_3$). ^{31}P NMR (202 MHz, Benzene-d_6) δ 38.5(m) ppm. ^{13}C NMR (125 MHz, Benzene-d_6) δ 150.2, 149.9, 130.0, 127.2, 115.4, 46.2, 40.0, 32.1, 25.9, 21.7, 10.1 ppm.

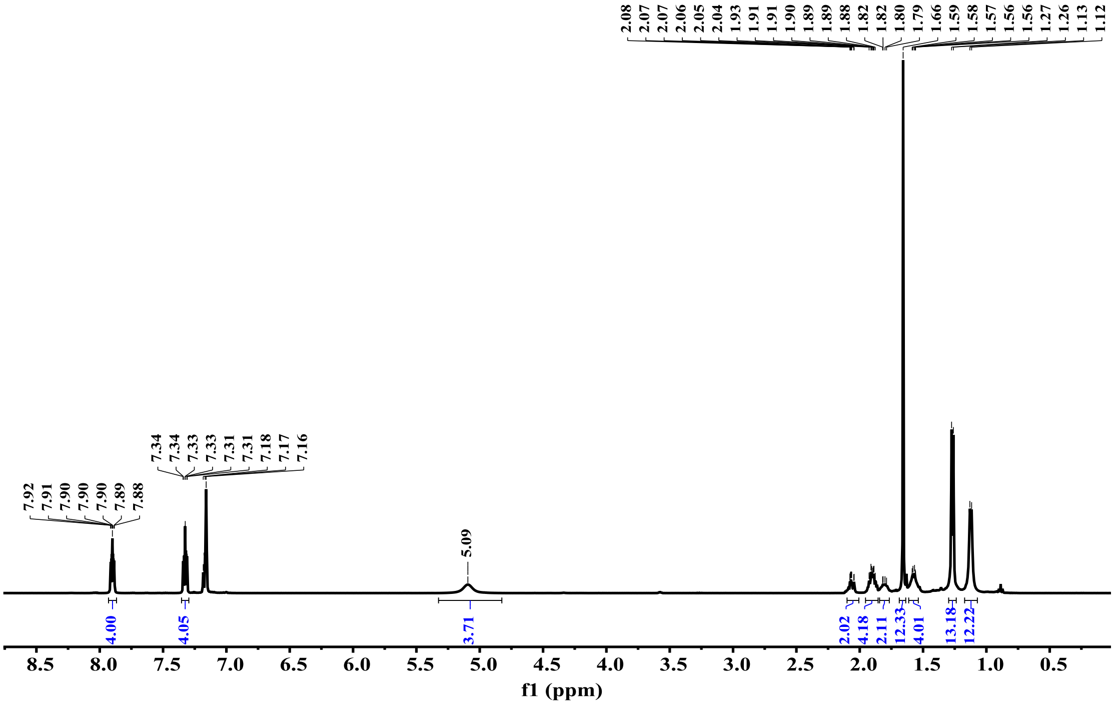

Supplementary Fig.25. ^{1}H NMR spectrum of μ^{Hex}[P(NI^{i}Pr)Ph]$_2$ (Benzene-d_6, 500 MHz).

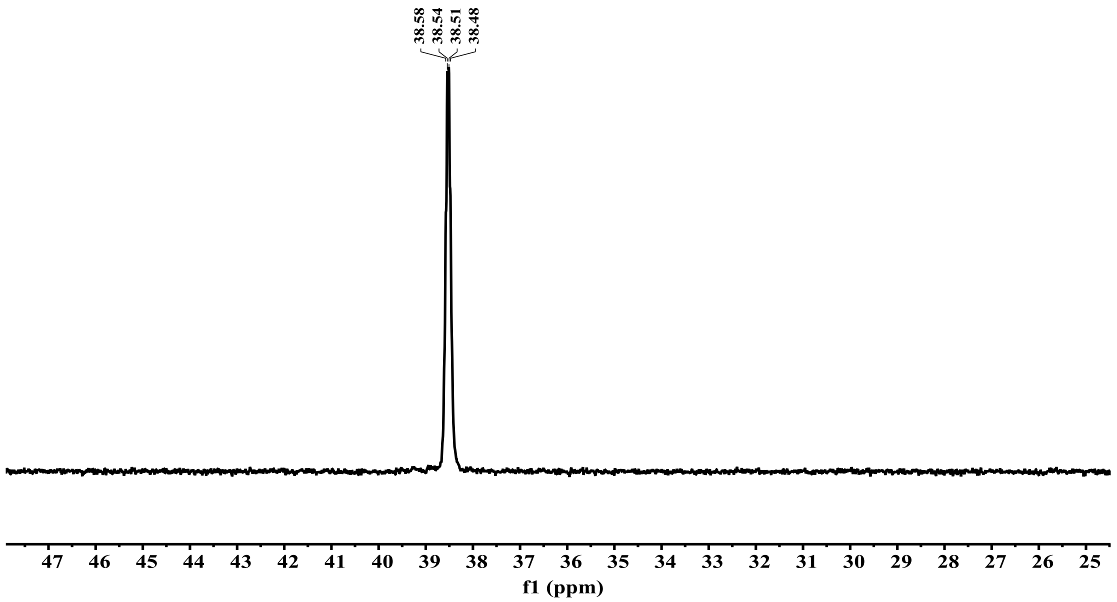

Supplementary Fig.26. ^{31}P NMR spectrum of μ^{Hex}[P(NI^{i}Pr)Ph]$_2$ (Benzene-d_6, 202 MHz).

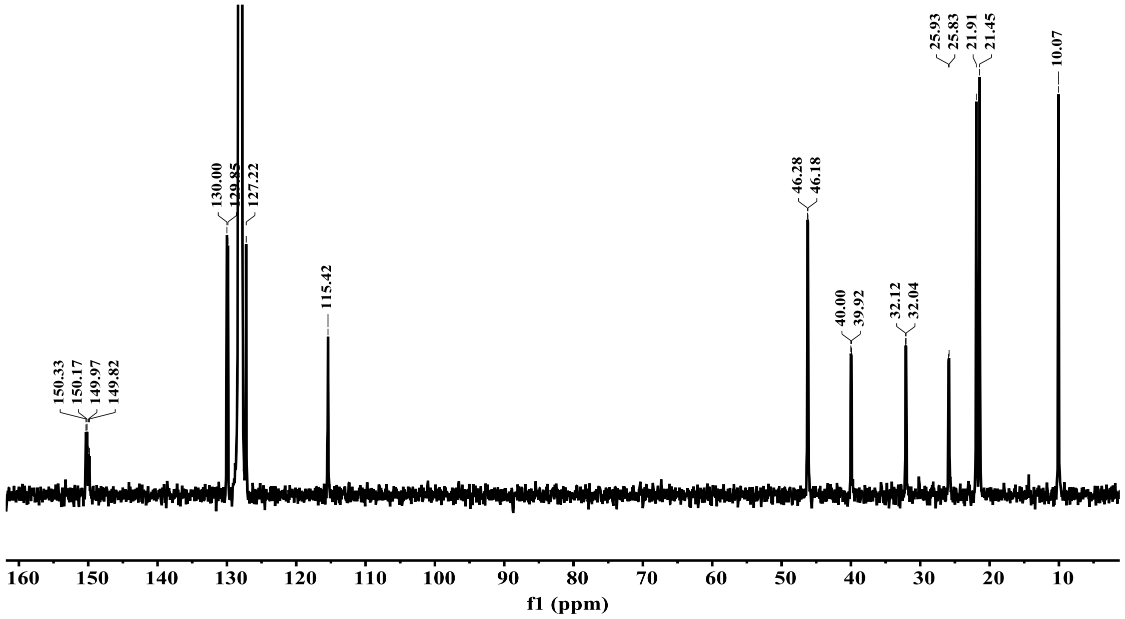

Supplementary Fig.27. ^{13}C NMR spectrum of μ^{Hex}[P(NI^{i}Pr)Ph]$_2$ (Benzene-d_6, 125 MHz).

Supplementary Methods 3 - Kinetic studies of bisfunctional organophosphorus superbases with different bridges.

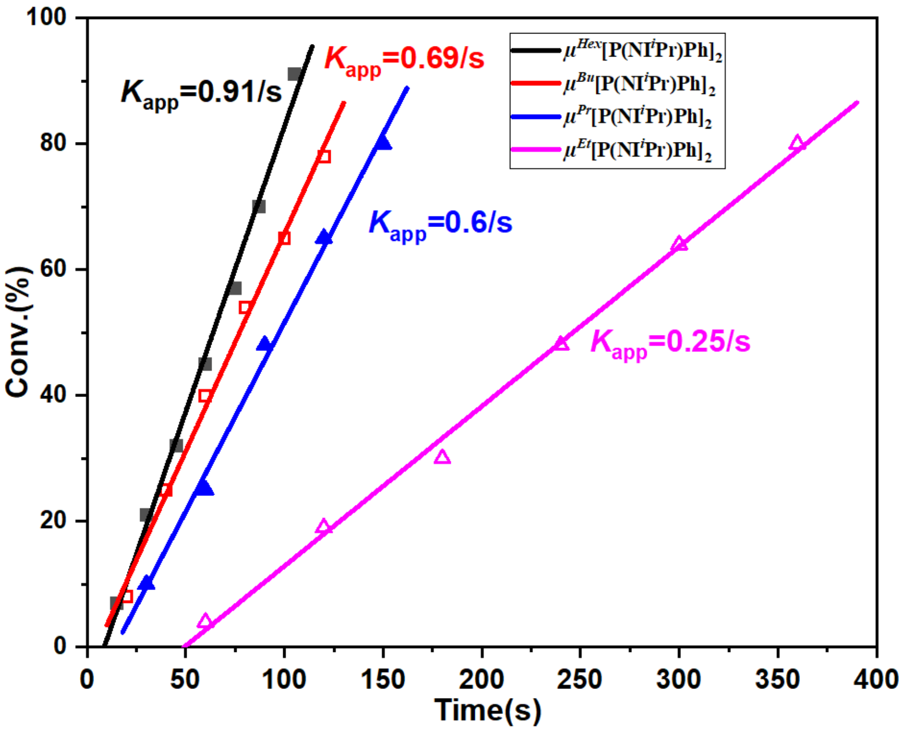

Supplementary Fig.28. The rate curves obtained for MMA polymerization catalyzed by LPs composed of $(BHT)_2AlMe$ and bisphosphines with different bridges.

Supplementary Methods 4 - Stoichiometric NMR Reaction

Supplementary Methods 4.1 - Stoichiometric NMR reaction of μ^{Hex}[P(NI^{i}Pr)Ph]$_2$ with $(BHT)_2AlMe$ in a 1:2 ratio

A Teflon-valve-sealed J. Young-type NMR tube was charged with μ^{Hex}[P(NI^{i}Pr)Ph]$_2$ (6.9 mg, 0.01 mmol) and 0.3 mL of C_6D_6, then added with a solution of $(BHT)_2AlMe$ (9.6 mg, 0.02 mmol, 0.3 mL C_6D_6) via pipette at ambient temperature. The mixture was allowed to react for 30 min before analysis by NMR. There is no reaction observed between μ^{Hex}[P(NI^{i}Pr)Ph]$_2$ and $(BHT)_2AlMe$.

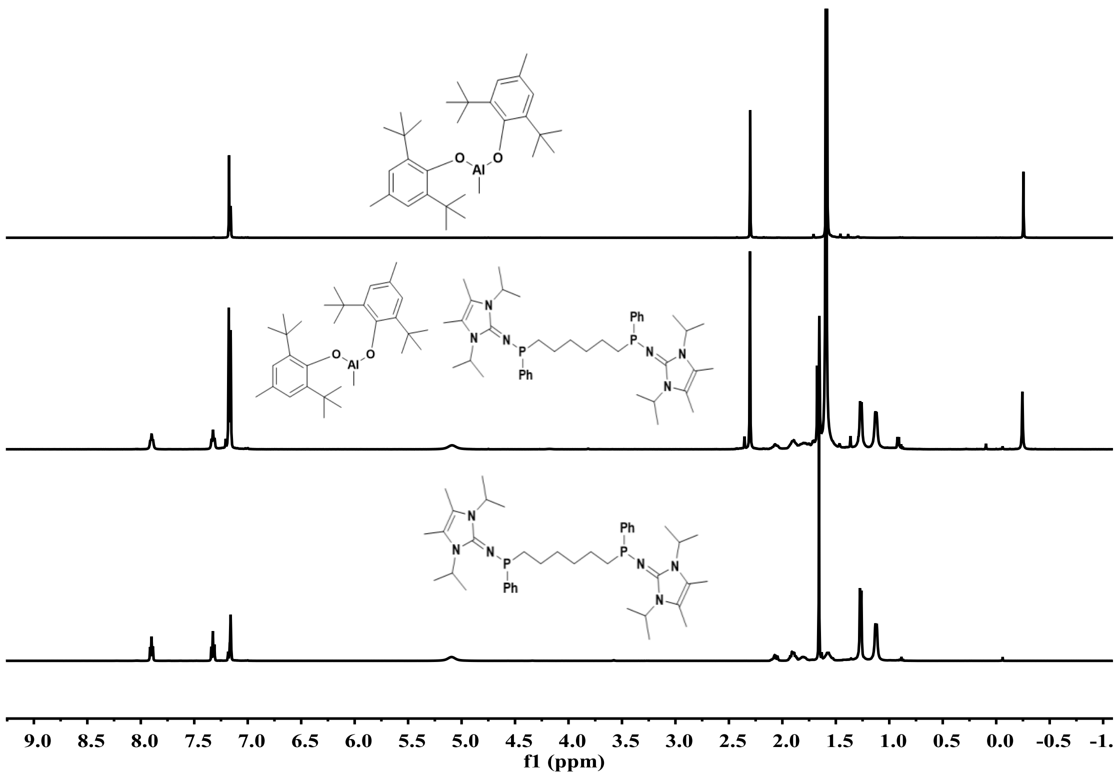

Supplementary Fig.29. Overlay of ^{1}H NMR spectra of (BHT)$_2$AlMe (top), reaction of μ^{Hex}[P(NI^{i}Pr)Ph]$_2$ with (BHT)$_2$AlMe (middle) and μ^{Hex}[P(NI^{i}Pr)Ph]$_2$ (bottom) (500 MHz, Benzene-d_6)

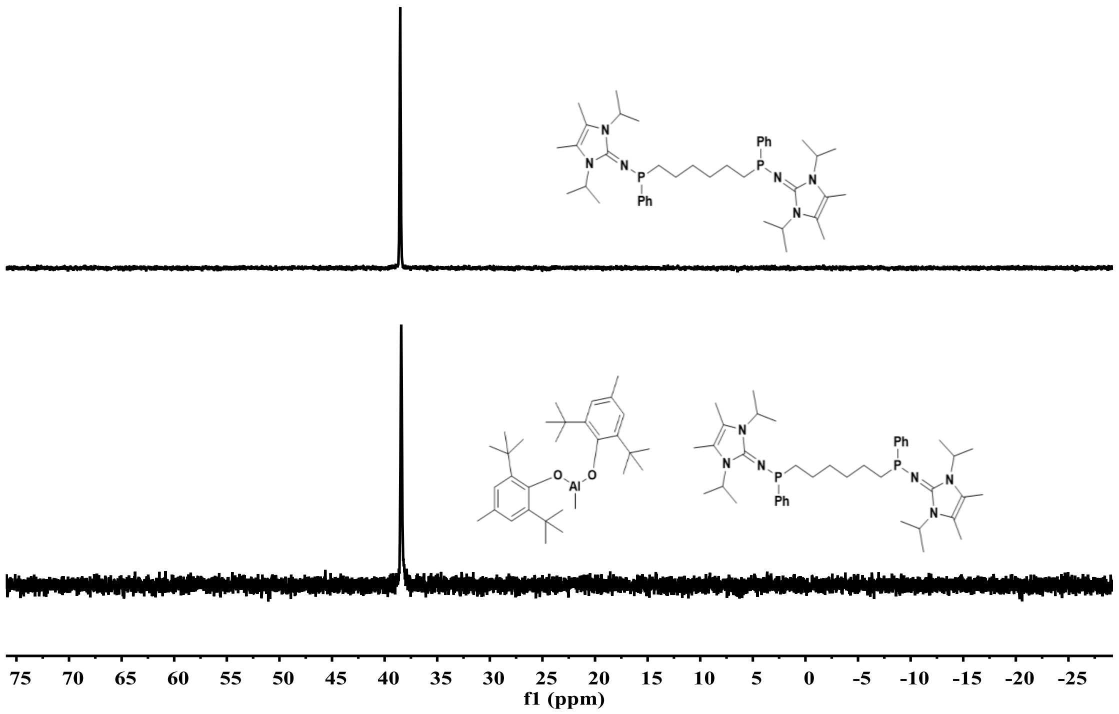

Supplementary Fig.30. Overlay of ^{31}P NMR spectra of μ^{Hex}[P(NI^{i}Pr)Ph]$_2$ (top) and the reaction of μ^{Hex}[P(NI^{i}Pr)Ph]$_2$ with (BHT)$_2$AlMe (bottom) (202 MHz, Benzene-d_6).

Supplementary Methods 4.2 - Stoichiometric NMR reaction of μ^{Bu}[P(NI^{i}Pr)Ph]$_2$ with (BHT)$_2$AlMe·MMA in a 1:2 ratio

In an argon-filled glovebox, a Teflon-valve-sealed J. Young-type NMR tube was charged with μ^{Bu}[P(NI^{i}Pr)Ph]$_2$ (6.61 mg, 0.01 mmol) in 0.3 mL of C_6D_6, then slowly added with 0.3 mL C_6D_6 solution of (BHT)$_2$AlMe·MMA (11.62 mg, 0.02 mmol) via pipet at RT. The mixture was allowed to react for 15 min before the NMR spectra were recorded, which showed the clean formation of the biszwitterion **1** as two isomers A (major) and B (minor) about in a 1:3 ratio and some distinguish impurities. **1A**: ^{1}H NMR (500 MHz, Benzene-d_6) δ 7.54 - 7.49 (m, 4H, Ph-H), 7.27 (s, 8H, Ar-H), 7.04 - 7.01 (m, 6H, Ph-H), 4.21 - 4.15 (m, 4H, N*CH*(CH$_3$)$_2$), 3.80 (d, *J* = 15 Hz, 4H, P*CH$_2$*), 3.71 (s, 3H, O*Me*), 3.18-3.09 (m, 8H, P*CH$_2$*CH$_2$), 2.40-2.36 (m, 8H, PCH$_2$*CH$_2$*), 2.40 (s, 12H, Ar-*Me*), 1.84 (s, 36H, ^{t}*Bu*Ar), 1.63 (d, *J* = 5 Hz, 6H, =C*Me*), 1.42 (s, 12H, *Me*C=C*Me*), 0.83(m, 24H, NCH(*CH$_3$*)$_2$), 0.01(s, 6H, AlMe). ^{31}P NMR (202 MHz, Benzene-d_6) δ 19.8 ppm (m). **1B**: ^{1}H NMR (500 MHz, Benzene-d_6) δ 7.42 - 7.37 (m, 4H, Ph-H), 7.28 (s, 8H, Ar-H), 7.12 - 7.09 (m, 6H, Ph-H), 4.09 - 4.02 (m, 4H, N*CH*(CH$_3$)$_2$), 3.40 (d, *J* = 15 Hz, 4H, P*CH$_2$*), 3.26 (s, 3H, O*Me*), 2.85-2.70 (m, 8H, P*CH$_2$*CH$_2$), 2.40-2.36 (m, 8H, PCH$_2$*CH$_2$*), 2.38 (s, 12H, Ar-*Me*), 1.82 (s, 36H, ^{t}*Bu*Ar), 1.78 (d, *J* = 5 Hz, 6H, =C*Me*), 1.44 (s, 12H, *Me*C=C*Me*), 0.89(m, 24H, NCH(*CH$_3$*)$_2$), -0.02(s, 6H, AlMe). ^{31}P NMR (202 MHz, Benzene-d_6) δ 19.8 ppm (m).

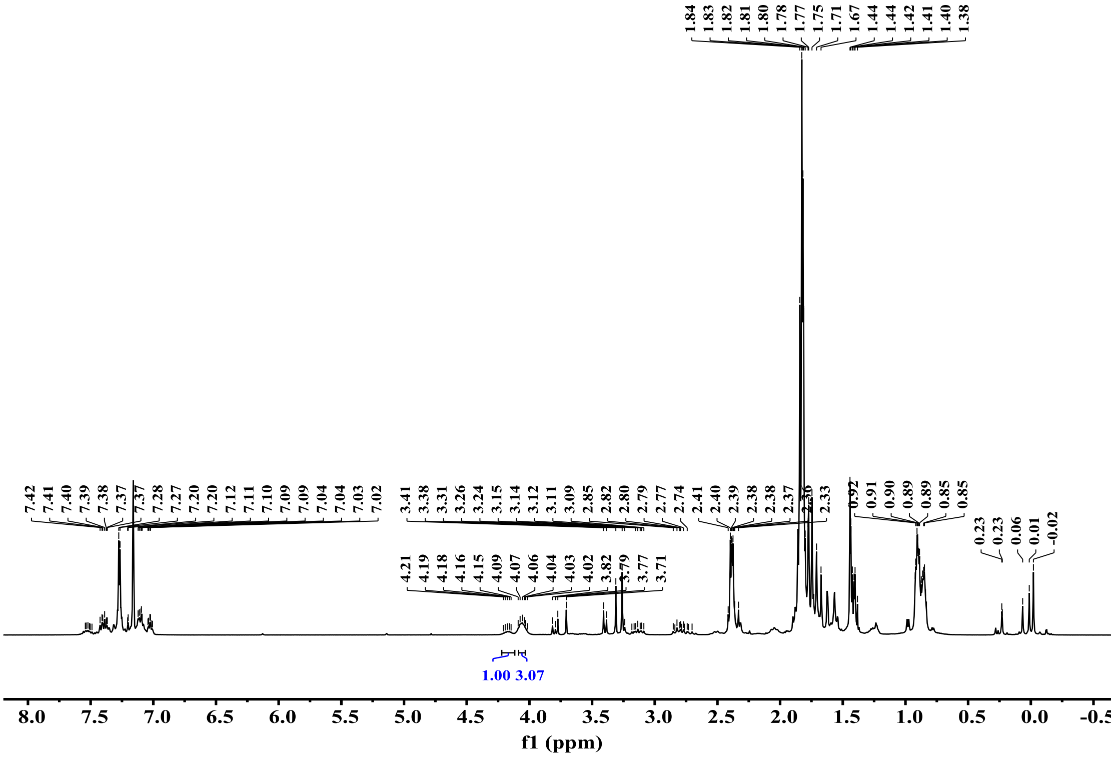

Supplementary Fig.31. ^{1}H NMR spectrum obtained for the reaction of μ^{Bu}[P(NI^{i}Pr)Ph]$_2$ with

$(BHT)_2AlMe\cdot MMA$ (500 MHz, Benzene-d_6).

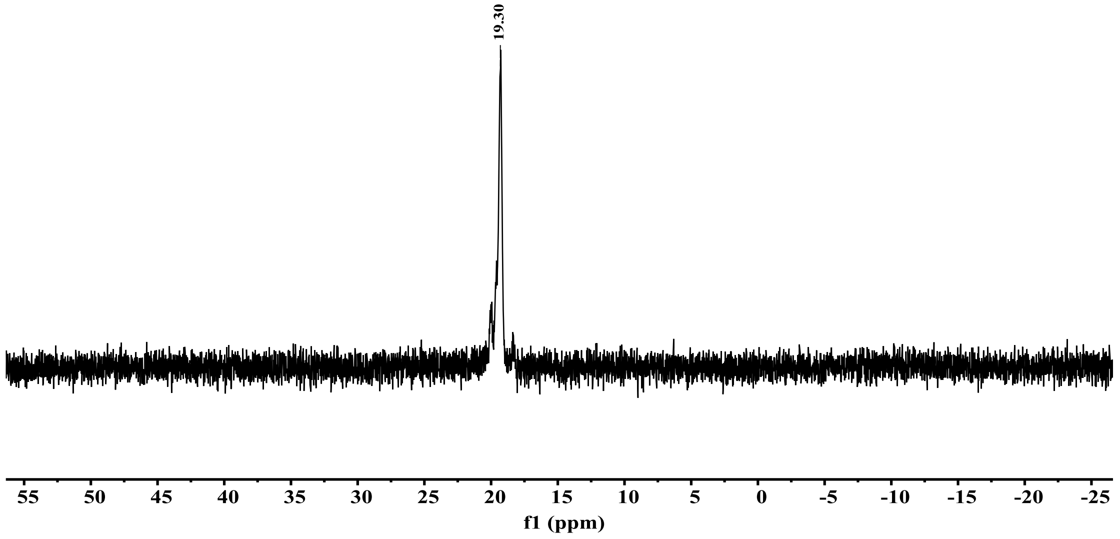

Supplementary Fig.32. ^{31}P NMR spectrum obtained for the reaction of $\mu^{Bu}[P(NI^{i}Pr)Ph]_2$ with $(BHT)_2AlMe\cdot MMA$. (202 MHz, Benzene-d_6).

Supplementary Methods 4.3 - Stoichiometric NMR reaction of $\mu^{Hex}[P(NI^{i}Pr)Ph]_2$ with $(BHT)_2AlMe\cdot MMA$ in a 1:2 ratio

In an argon-filled glovebox, a Teflon-valve-sealed J. Young-type NMR tube was charged with 6.89 mg (0.01 mmol) of $\mu^{Hex}[P(NI^{i}Pr)Ph]_2$ and 0.3 mL of C_6D_6. A 0.3 mL C_6D_6 solution of $(BHT)_2AlMe\cdot MMA$ (11.62 mg, 0.02 mmol) was slowly added to this tube via pipet at RT. The mixture was allowed to react for 15 min before the NMR spectra were recorded, which showed the clean formation of the biszwitterion **2** as two isomers A (major) and B (minor) about in a 1:2 ratio and some distinguish impurities. **2A**: ^{1}H NMR (500 MHz, Benzene-d_6) δ 7.46 - 7.42 (m, 4H, Ph-H), 7.27 (s, 8H, Ar-H), 7.11 - 7.09 (m, 6H, Ph-H), 4.09 - 4.04 (m, 4H, N*CH*(CH_3)$_2$), 3.45 (d, J = 15 Hz, 4H, P*CH$_2$*), 3.44 (s, 3H, O*Me*), 2.96-2.90 (m, 8H, P*CH$_2$*CH_2CH_2), 2.52-2.43 (m, 8H, PCH_2*CH$_2$*CH_2), 2.38 (s, 12H, Ar-*Me*), 1.85 (s, 36H, ^{t}*Bu*Ar), 1.67 (d, J = 5 Hz, 6H, =C*Me*), 1.44 (s, 12H, *Me*C=C*Me*), 0.97 (m, 8H, PCH_2CH_2*CH$_2$*), 0.88(m, 24H, NCH(*CH$_3$*)$_2$), 0.10(s, 6H, AlMe). ^{31}P NMR (202 MHz, Benzene-d_6) δ 19.9 ppm (m). **2B**: ^{1}H NMR (500 MHz, Benzene-d_6) δ 7.42 - 7.38 (m, 4H, Ph-H), 7.27 (s, 8H, Ar-H), 7.22 - 7.19 (m, 6H, Ph-H), 4.22 - 4.15 (m, 4H, N*CH*(CH_3)$_2$), 3.80 (d, J = 15 Hz, 4H, P*CH$_2$*), 3.78 (s, 3H, O*Me*), 3.14-3.09 (m, 8H, P*CH$_2$*CH_2CH_2), 2.38 (s, 12H, Ar-*Me*), 2.24-2.10 (m, 8H, PCH_2*CH$_2$*CH_2), 1.84 (s, 36H, ^{t}*Bu*Ar), 1.76 (d, J = 5 Hz, 6H, =C*Me*), 1.43 (s, 12H, *Me*C=C*Me*), 0.98 (m, 8H, PCH_2CH_2*CH$_2$*), 0.87(m, 24H, NCH(*CH$_3$*)$_2$), 0.13(s, 6H, AlMe). ^{31}P NMR (202 MHz, Benzene-d_6) δ 19.9 ppm (m).

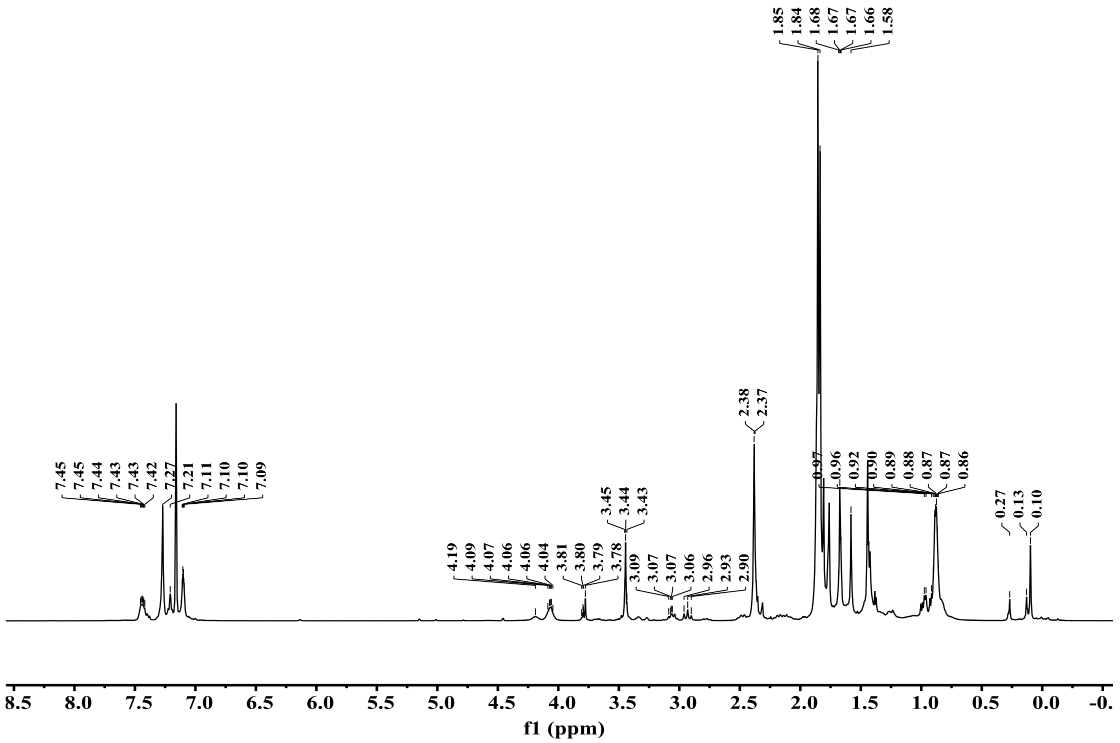

Supplementary Fig.33. ^{1}H NMR spectrum for the reaction of μ^{Hex}[P(NI^{i}Pr)Ph]$_2$ with (BHT)$_2$AlMe·MMA (500 MHz, Benzene-d_6).

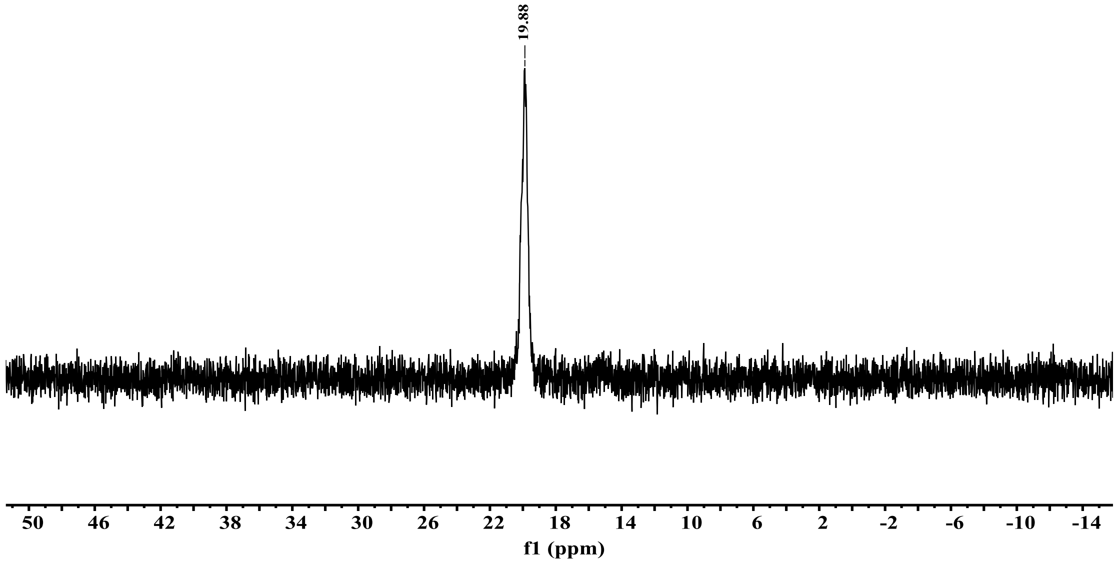

Supplementary Fig.34. ^{31}P NMR spectrum for the reaction of μ^{Hex}[P(NI^{i}Pr)Ph]$_2$ with (BHT)$_2$AlMe·MMA. (202 MHz, Benzene-d_6).

Supplementary Methods 5 - Select polymerization results

Supplementary Table 1. Polymerization by μ^{Hex}[P(NI^{i}Pr)Ph]$_2$/(BHT)$_2$AlMe LP^{a}

Run	LB	LA	M	[M]:[LA]:[LB]	T (min)	Conv.[b] (%)	M_w[c] (kg·mol^{-1})	$Đ$[c]	I^{*d} (%)
1	μ^{Hex}[P(NI^{i}Pr)Ph]$_2$	(BHT)$_2$AlMe	MMBL	400:4:1	2	100	73.6	1.28	78
2	μ^{Hex}[P(NI^{i}Pr)Ph]$_2$	(BHT)$_2$AlMe	MMBL	400:4:1	2	100			
			MMBL	400:4:1	2	100	94.0	1.07	
3	μ^{Hex}[P(NI^{i}Pr)Ph]$_2$	(BHT)$_2$AlMe	MMBL	400:4:1	2	100			
			MBL	400:4:1	2	100	94.0	1.04	
4[e]	μ^{Hex}[P(NI^{i}Pr)Ph]$_2$	(BHT)$_2$AlMe	MMA	400:4:1	3	100			
			MMBL	400:4:1	2	100	(84.2)	(1.21)	

[a] Conditions: polymerization was performed in a 400:4:1 [monomer]: [(BHT)$_2$AlMe]:[μ^{Hex}[P(NI^{i}Pr)Ph]$_2$] ratio in dichloromethane at RT; $[M]_0$ = 0.936 M. [b] Monomer conversions measured by ^{1}H NMR spectroscopy. [c] Absolute molecular weight (M_w) measured by GPC using a light scattering detector. [d] Initiator efficiency (I^*)% = M_n(calcd)/M_n(exptl) × 100, where M_n(calcd) = [MW(MMBL)] × ([MMBL]$_0$/[I]$_0$) (conversion) + MW of chain-end groups. [e] toluene used as solvent. M_n and $Đ$ (in the parenthesis) determined by GPC relative to PMMA standards in DMF.

Supplementary Table 2. Mechanical properties of ABA-type triblock TPEs[a]

Run[a]	Monomers	f_{hard}[b]	Conv. (%)[c]	σ (MPa)[d]	ε (%)[d]	M_n[e] (kg·mol^{-1})	$Đ$[e]
1	100MMBL-1600EEMA-100MMBL	0.11	100	6.46±0.19	728±53	201	1.22
2	200MMBL-1600EEMA-200MMBL	0.2	100	7.67±0.42	719±32	204	1.26
3	300MMBL-1600EEMA-300MMBL	0.27	100	8.42±0.49	660±39	276	1.26
4	400MMBL-1600EEMA-400MMBL	0.33	100	10.8±0.23	598±15	302	1.30
5	100MMA-1600EEMA-100MMA	0.11	100	5.27±0.19	524±15	222	1.19
6	200MMA-1600EEMA-200MMA	0.2	100	6.35±0.27	478±47	242	1.19
7	300MMA-1600EEMA-300MMA	0.27	100	8.38±0.14	395±24	260	1.19
8	400MMA-1600EEMA-400MMA	0.33	100	8.59±0.11	386±21	293	1.21

[a] Polymerization conditions: All ABA block copolymers synthesis by μ^{Hex}[P(NI^{i}Pr)Ph]$_2$/(BHT)$_2$AlMe FLP system through sequential monomer addition method in one pot. [b] Fraction of hard segment calculated from the molar quantity value of each segment and the overall molar quantity value. [c] Monomer conversions measured by ^{1}H NMR spectroscopy. [d] Stress at break (σ) and strain at break (ε) determined by stress−strain tensile testing, [e] M_n and $Đ$ determined by GPC relative to PMMA standards in DMF.

Supplementary Table 3. Polymerization results by different LPs[a]

Run	I	LA	M	[M]:[LA]:[I]	T(min)	Conv.[b] (%)	M_n[c] (kg·mol^{-1})	$Đ$[c]
1	μ^{Hex}[P(NI^{i}Pr)Ph]$_2$	Al(C_6F_5)$_3$	MMA	400:4:1	30	100	42.5	1.18
2[d]	μ^{Hex}[P(NI^{i}Pr)Ph]$_2$	Al(C_6F_5)$_3$	MMA	400:4:1	30	100		
			MMA	400:4:1	5	100	124/42.1 (45.4%/54.6%)	1.09/1.12
3	biszwitterion 1	-	MMA	400:0:1	960	0		
4[e]	biszwitterion 1	(BHT)$_2$AlMe	MMA	400:2:1	4	100	34.3	1.17
5	biszwitterion 1	(BHT)$_2$AlMe	MMA	400:2:1	4	100	39.4	1.14

[a] Conditions: carried out at RT in toluene (TOL), [M] = [MMA]$_0$ = 0.936 M, I = Initiator, the LB or biszwitterion1 as initiator. [b] Monomer conversions measured by ^{1}H NMR. [c] M_n and $Đ$ determined by GPC relative to PMMA standards in DMF. [d] chain extension experiment by μ^{Hex}[P(NI^{i}Pr)Ph]$_2$/Al(C_6F_5)$_3$ LP. [e] Premixed biszwitterion 1 and monomer at RT for 16 hours, then add 2 equiv. LA to initiate polymerization.

Supplementary Methods 6 - The optical properties of biorenewable PMMBL-based TPEs

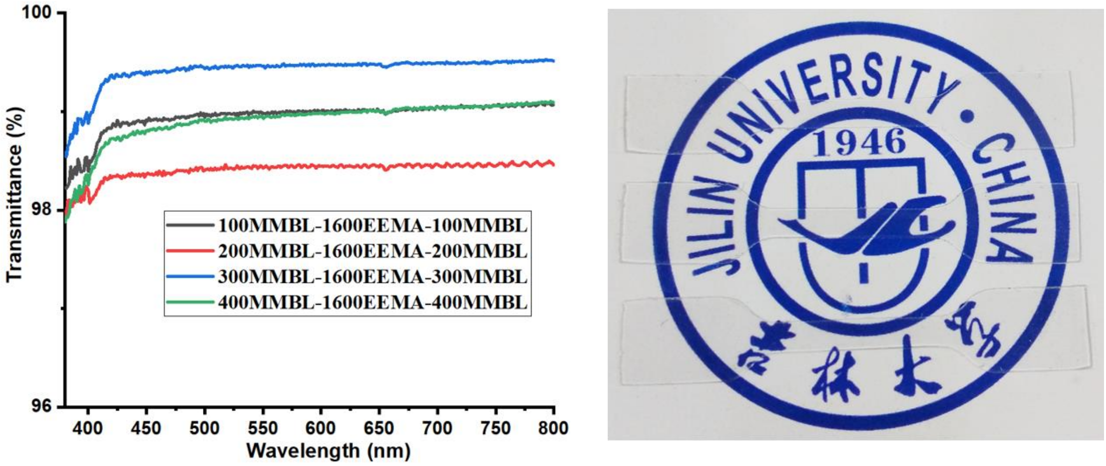

Supplementary Fig.35. The optical properties of biorenewable PMMBL-based TPEs.

Supplementary Methods 7 - Plots of M_n and $Đ$ of PMMBL vs MMBL conversion

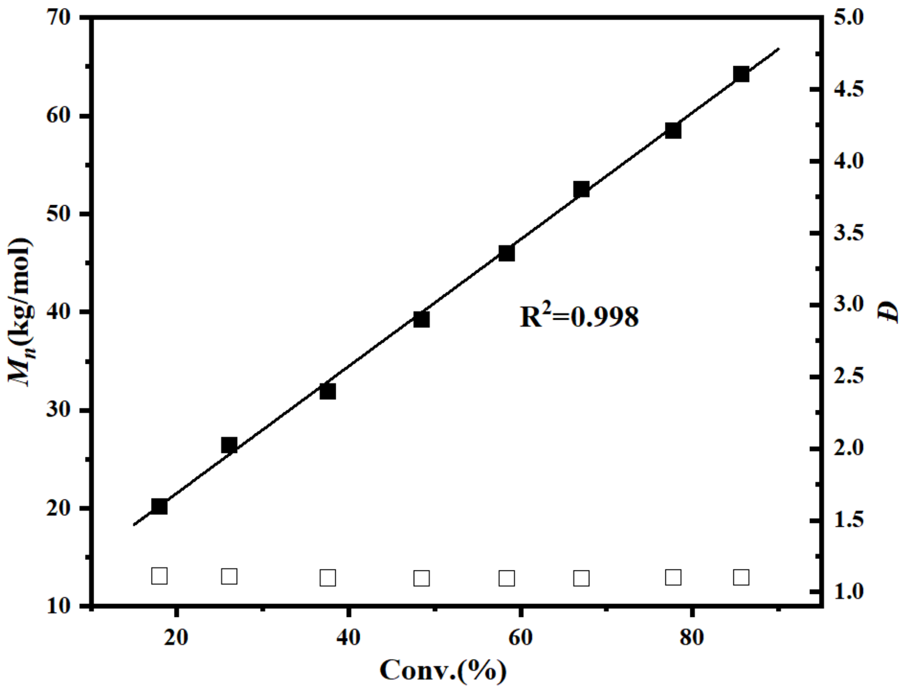

Supplementary Fig.36. Plots of M_n and $Đ$ of PMMBL vs MMBL conversion catalyzed by FLP at room temperature. Conditions: [MMBL]/[μ^{Hex}[P(NI^{i}Pr)Ph]$_2$]/[(BHT)$_2$AlMe] = 800/1/4 in dichloromethane at RT, [MMBL]$_0$ = 0.936 M.

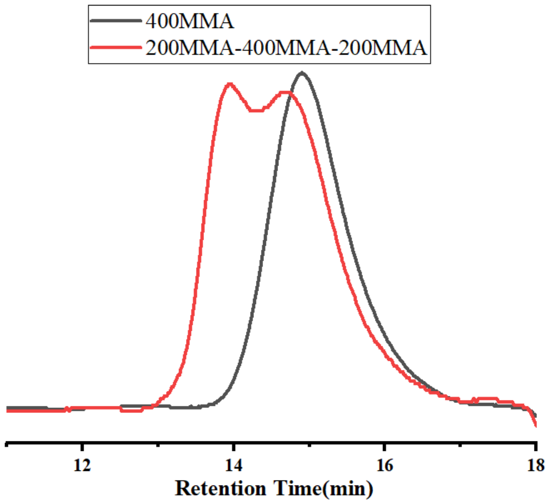

Supplementary Fig.37. GPC traces for PMMA samples obtained from chain extension experiments by μ^{Hex}[P(NI^{i}Pr)Ph]$_2$/Al(C_6F_5)$_3$.

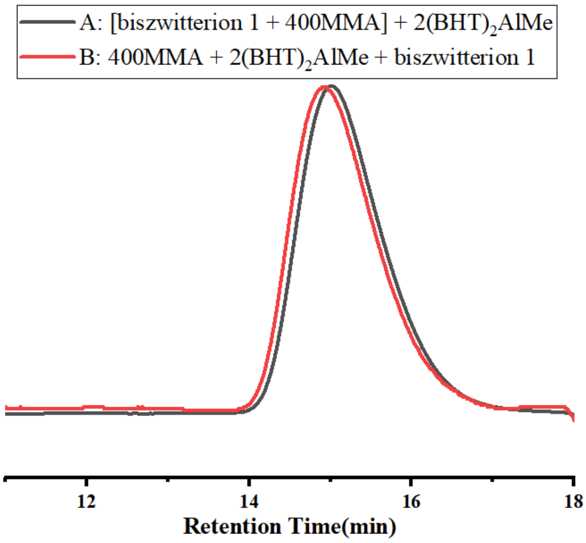

Supplementary Fig.38. GPC traces of PMMA obtained from the polymerization performed using different procedures. A: premixed biszwitterion 1 and monomer at RT for 16 hours, then add 2 equiv. LA to initiate polymerization; B: premixed monomer and 2 equiv. LA, then immediately add biszwitterion 1 to initiate polymerization

Supplementary Methods 8 - X-ray diffraction data

Single crystals were quickly covered with a layer of Paratone-N oil (Exxon, dried and degassed at 120°C/10^{-6} Torr for 24 h) after decanting the mother liquor. A crystal was then mounted on a thin glass fiber and transferred into the cold nitrogen stream of a Bruker

APEX-II CCD diffractometer. The structures were solved by direct methods and refined using the Bruker SHELXTL program library by full-matrix least squares on *F2* for all reflections (*SHELXTL*, Version 6.12; Bruker Analytical X-ray Solutions: Madison, WI, 2001). The structure was refined by full-matrix least-squares on *F*2 for all reflections. All non-hydrogen atoms were refined with anisotropic displacement parameters, whereas hydrogen atoms were included in the structure factor calculations at idealized positions (Sheldrick, G. M. *Acta Crystallogr., Sect. A.* **1990**, *46*, 467–473 & **2008**, *64*, 112–122.).

Supplementary Table 4. Crystal data and structure refinement for μ^{Bu}[P(NI^{i}Pr)Ph]$_2$, μ^{Hex}[P(NI^{i}Pr)Ph]$_2$ and biszwitterionic species.

Compound	μ^{Bu}[P(NI^{i}Pr)Ph]$_2$	μ^{Hex}[P(NI^{i}Pr)Ph]$_2$	biszwitterion 1
CCDC #	2064107	2064108	2064109
empirical formula	$C_{38}H_{58}N_6P_2$	$C_{40}H_{62}N_6P_2$	$Al_2P_2N_6O_8C_{146}H_{208}$
formula weight	660.84	688.89	2291.07
crystal system	monoclinic	monoclinic	monoclinic
space group	P2$_1$/c	P2$_1$/c	P2$_1$/c
a/Å	9.6494(9)	12.7377(10)	12.2897(10)
b/Å	11.1111(10)	13.7503(9)	17.8164(17)
c/Å	17.9171(15)	11.3067(9)	31.512(3)
α/ deg	90	90	90
β/ deg	90.864(3)	92.521(3)	98.305(4)
γ/ deg	90	90	90
volume/Å^3	1920.8(3)	1978.4(3)	6827.4(10)
Z	2	2	2
ρ_{calc}g/cm^3	1.143	1.156	1.114
μ/mm^{-1}	0.147	0.145	0.101
F(000)	716.0	748	2492.0
crystal size/mm^3	0.12 × 0.11 × 0.1	0.19 × 0.17 × 0.16	0.1 × 0.09 × 0.08
radiation(MoKα, λ)	0.71073	0.71073	0.71073
θ range (deg)	2.796 to 26.749	2.785 to 26.739	2.54 to 26.775
limiting indices	$-12 \le h \le 12$, $-14 \le k \le 14$, $-22 \le l \le 22$	$-16 \le h \le 16$, $-17 \le k \le 17$, $-14 \le l \le 14$	$-15 \le h \le 14$, $-22 \le k \le 22$, $-39 \le l \le 39$
reflns collected	33365	60097	99196
independent reflns	4075 [R_{int} = 0.1491]	4207 [R_{int} = 0.0294]	14502 [R_{int} = 0.0914]
absorption correction	None	None	None
data/restraints/parameters	4075/299/328	4207 / 0 / 223	14502/6/763
goodness-of-fit on F^2	1.017	1.076	1.067
final R indexes [I ≥ 2σ (I)] [a]	R_1 = 0.0828, wR_2 = 0.1659	R_1 = 0.0523, wR_2 = 0.1367	R_1 = 0.0803, wR_2 = 0.1846
final R indexes [all data][a]	R_1 = 0.1596, wR_2= 0.2015	R_1 = 0.0546, wR_2 = 0.1384	R_1 = 0.1109, wR_2 = 0.2009
peak/hole / e Å^{-3}	0.52/-0.36	1.081 / -0.418	1.72/-0.69

[a] $R1 = \Sigma||F_o|-|F_c||/\Sigma|F_o|$; $wR2 = \{\Sigma[w(F_o^2-F_c^2)^2]/\Sigma[w(F_o^2)^2]\}^{1/2}$

Supplementary Table 5. Bond Lengths [Å] and Angles [°] for μ^{Bu}[P(NI^{i}Pr)Ph]$_2$.

P1-N1	1.655(5)	C13-C15	1.350(5)
P1-C2	1.855(5)	C13-N2A	1.336(15)
P1-C3	1.881(3)	C15-C16	1.491(5)
N1-C9	1.334(6)	C17-C18	1.502(5)
N2-C9	1.367(6)	C17-C19	1.510(5)
N2-C10	1.489(6)	C1A-C1A^1	1.557(8)
N2-C13	1.429(6)	C1A-C2A	1.499(11)
N3-C9	1.388(5)	C2A-P1A	1.760(14)
N3-C15	1.403(5)	P1A-C3A	1.910(11)
N3-C17	1.467(4)	P1A-N1A	1.621(13)
N3-C9A	1.361(14)	C3A-C8A	1.3900
C1-C1^1	1.711(12)	C3A-C4A	1.3900
C1-C2	1.503(7)	C8A-C7A	1.3900
C3-C4	1.3900	C7A-C6A	1.3900
C3-C8	1.3900	C6A-C5A	1.3900
C4-C5	1.3900	C5A-C4A	1.3900
C5-C6	1.3900	N1A-C9A	1.252(14)
C6-C7	1.3900	C9A-N2A	1.368(15)
C7-C8	1.3900	N2A-C10A	1.482(15)
C10-C11	1.512(7)	C12A-C10A	1.533(18)
C10-C12	1.524(10)	C10A-C11A	1.516(17)
C13-C14	1.496(6)		
N1-P1-C2	97.8(3)	N2A-C13-C14	121.0(8)
N1-P1-C3	104.4(2)	N2A-C13-C15	109.7(8)
C2-P1-C3	95.7(2)	N3-C15-C16	123.5(3)
C9-N1-P1	130.1(4)	C13-C15-N3	106.7(3)
C9-N2-C10	117.3(5)	C13-C15-C16	129.5(4)
C9-N2-C13	110.2(4)	N3-C17-C18	112.8(3)
C13-N2-C10	131.5(5)	N3-C17-C19	113.4(3)
C9-N3-C15	111.2(3)	C18-C17-C19	111.4(3)
C9-N3-C17	121.4(3)	C1A-C2A-P1A	122.0(15)
C15-N3-C17	127.3(3)	C2A-P1A-C3A	92.0(10)
C9A-N3-C15	104.6(7)	N1A-P1A-C2A	106.0(9)
C9A-N3-C17	126.0(7)	N1A-P1A-C3A	103.2(7)
C2-C1-C1^1	105.2(6)	C8A-C3A-P1A	116.9(7)
C1-C2-P1	110.5(4)	C8A- C3A-C4A	120.0
C4-C3-P1	122.4(2)	C4A- C3A-P1A	122.5(7)
C4-C3-C8	120.0	C7A- C8A-C3A	120.0
C8-C3- P1	117.5(2)	C8A- C7A-C6A	120.0
C5-C4-C3	120.0	C5A- C6A-C7A	120.0
C4-C5-C6	120.0	C6A- C5A-C4A	120.0
C7-C6-C5	120.0	C5A- C4A-C3A	120.0
C8-C7-C6	120.0	C9A- N1A-P1A	120.5(14)
C7-C8-C3	120.0	N3- C9A-N2A	109.9(11)
N1-C9-N2	124.6(5)	N1A-C9A-N3	136.4(15)
N1-C9-N3	130.8(5)	N1A-C9A-N32A	110.5(14)
N2-C9-N3	104.5(4)	C13-N2A- C9A	106.4(11)
N2-C10-C11	111.0(5)	C13-N2A-C10A	110.9(13)
N2-C10-C12	112.7(7)	C9A-N2A-C10A	142.2(15)
C11-C10-C12	112.7(6)	N2A-C10A-C12A	99(2)
N2-C13-C14	124.5(4)	N2A-C10A-C11A	105.5(17)

C15-C13-N2	107.2(4)	C11A-C10A-C12A	112(2)
C15-C13-C14	128.0(4)		

[1] -X,1-Y,1-Z

Supplementary Table 6. Bond Lengths [Å] and Angles [°] for μ^{Hex}[P(N^{i}Pr)Ph]$_2$.

C(1)-C(2)	1.528(3)	C(1)-P(1)	1.8462(18)
C(2)-C(3)	1.530(2)	C(3)-C(3)#1	1.518(4)
C(4)-N(1)	1.300(2)	C(4)-N(3)	1.378(2)
C(4)-N(2)	1.385(2)	C(5)-C(6)	1.347(3)
C(5)-N(2)	1.406(2)	C(5)-C(13)	1.497(3)
C(6)-N(3)	1.400(2)	C(6)-C(14)	1.495(3)
C(7)-N(2)	1.471(2)	C(7)-C(9)	1.525(2)
C(7)-C(8)	1.528(2)	C(10)-N(3)	1.476(2)
C(10)-C(11)	1.519(3)	C(10)-C(12)	1.524(3)
C(15)-C(16)	1.389(3)	C(15)-C(20)	1.397(3)
C(15)-P(1)	1.8439(19)	C(16)-C(17)	1.398(3)
C(17)-C(18)	1.387(3)	C(18)-C(19)	1.377(3)
C(19)-C(20)	1.400(3)	N(1)-P(1)	1.6731(16)
C(2)-C(1)-P(1)	115.53(13)	C(1)-C(2)-C(3)	112.95(16)
C(3)#1-C(3)-C(2)	112.9(2)	N(1)-C(4)-N(3)	123.16(15)
N(1)-C(4)-N(2)	131.53(16)	N(3)-C(4)-N(2)	105.24(14)
C(6)-C(5)-N(2)	107.43(15)	C(6)-C(5)-C(13)	128.52(18)
N(2)-C(5)-C(13)	123.97(17)	C(5)-C(6)-N(3)	107.76(16)
C(5)-C(6)-C(14)	129.27(18)	N(3)-C(6)-C(14)	122.73(17)
N(2)-C(7)-C(9)	112.09(15)	N(2)-C(7)-C(8)	112.69(15)
C(9)-C(7)-C(8)	112.26(15)	N(3)-C(10)-C(11)	112.15(15)
N(3)-C(10)-C(12)	111.64(15)	C(11)-C(10)-C(12)	112.77(16)
C(16)-C(15)-C(20)	118.59(17)	C(16)-C(15)-P(1)	122.47(15)
C(20)-C(15)-P(1)	118.88(14)	C(15)-C(16)-C(17)	120.5(2)
C(18)-C(17)-C(16)	120.3(2)	C(19)-C(18)-C(17)	119.8(2)
C(18)-C(19)-C(20)	120.0(2)	C(15)-C(20)-C(19)	120.78(19)
C(4)-N(1)-P(1)	125.43(12)	C(4)-N(2)-C(5)	109.61(14)
C(4)-N(2)-C(7)	122.91(14)	C(5)-N(2)-C(7)	127.12(14)
C(4)-N(3)-C(6)	109.90(14)	C(4)-N(3)-C(10)	125.31(15)
C(6)-N(3)-C(10)	124.75(15)	N(1)-P(1)-C(15)	102.18(8)
N(1)-P(1)-C(1)	100.37(8)	C(15)-P(1)-C(1)	98.46(8)

[#1] -x+1,-y+1,-z+1

Supplementary Table 7. Bond Lengths [Å] and Angles [°] for biszwitterion 1.

Al(1)-O(4)	1.752(2)	Al(1)-O(3)	1.752(2)
Al(1)-O(1)	1.802(3)	Al(1)-C(25)	1.968(4)
C(1)-N(1)	1.352(5)	C(1)-N(3)	1.357(5)
C(1)-N(2)	1.373(4)	C(2)-C(3)	1.344(6)

C(2)-N(3)	1.407(5)	C(2)-C(5)	1.495(6)
C(3)-N(2)	1.409(5)	C(3)-C(4)	1.511(6)
C(6)-N(2)	1.472(5)	C(6)-C(8)	1.515(6)
C(6)-C(7)	1.526(6)	C(9)-N(3)	1.492(5)
C(9)-C(11)	1.518(5)	C(9)-C(10)	1.528(5)
C(12)-C(13)	1.538(5)	C(12)-P(1)	1.810(3)
C(13)-C(13)#1	1.543(7)	C(14)-C(15)	1.400(5)
C(14)-C(19)	1.408(5)	C(14)-P(1)	1.816(4)
C(15)-C(16)	1.389(5)	C(16)-C(17)	1.384(6)
C(17)-C(18)	1.393(6)	C(18)-C(19)	1.373(5)
C(20)-C(21)	1.502(5)	C(20)-P(1)	1.834(3)
C(21)-C(23)	1.338(5)	C(21)-C(22)	1.514(5)
C(23)-O(1)	1.323(4)	C(23)-O(2)	1.395(4)
C(24)-O(2)	1.418(5)	C(26)-O(3)	1.389(3)
C(26)-C(27)	1.3900	C(26)-C(31)	1.3900
C(27)-C(28)	1.3900	C(27)-C(32)	1.586(4)
C(28)-C(29)	1.3900	C(29)-C(30)	1.3900
C(29)-C(40)	1.545(4)	C(30)-C(31)	1.3900
C(31)-C(36)	1.573(3)	C(32)-C(34)	1.536(5)
C(32)-C(35)	1.543(5)	C(32)-C(33)	1.544(5)
C(36)-C(38)	1.539(5)	C(36)-C(39)	1.543(5)
C(36)-C(37)	1.544(5)	C(41)-O(4)	1.351(4)
C(41)-C(46)	1.427(4)	C(41)-C(42)	1.434(4)
C(42)-C(43)	1.400(5)	C(42)-C(47)	1.541(4)
C(43)-C(44)	1.384(5)	C(44)-C(45)	1.386(5)
C(44)-C(55)	1.519(4)	C(45)-C(46)	1.401(4)
C(46)-C(51)	1.548(4)	C(47)-C(50)	1.539(5)
C(47)-C(49)	1.543(5)	C(47)-C(48)	1.544(5)
C(51)-C(53)	1.528(5)	C(51)-C(54)	1.535(5)
C(51)-C(52)	1.540(4)	C(56)-C(57)	1.3900
C(56)-C(61)	1.3900	C(57)-C(58)	1.3900
C(58)-C(59)	1.3900	C(59)-C(60)	1.3900
C(60)-C(61)	1.3900	C(62)-C(67)	1.369(8)
C(62)-C(63)	1.381(8)	C(63)-C(64)	1.377(7)
C(64)-C(65)	1.372(7)	C(65)-C(66)	1.379(7)
C(66)-C(67)	1.369(8)	C(68)-C(69)	1.372(8)
C(68)-C(73)	1.373(7)	C(69)-C(70)	1.381(8)
C(70)-C(71)	1.365(8)	C(71)-C(72)	1.374(7)
C(72)-C(73)	1.385(7)	N(1)-P(1)	1.569(3)
O(4)-Al(1)-O(3)	113.19(11)	O(4)-Al(1)-O(1)	104.24(12)
O(3)-Al(1)-O(1)	105.90(12)	O(4)-Al(1)-C(25)	116.37(14)
O(3)-Al(1)-C(25)	107.57(14)	O(1)-Al(1)-C(25)	109.00(14)
N(1)-C(1)-N(3)	121.3(3)	N(1)-C(1)-N(2)	131.7(3)
N(3)-C(1)-N(2)	107.0(3)	C(3)-C(2)-N(3)	106.5(3)

C(3)-C(2)-C(5)	128.9(4)	N(3)-C(2)-C(5)	124.6(4)
C(2)-C(3)-N(2)	108.6(3)	C(2)-C(3)-C(4)	128.0(4)
N(2)-C(3)-C(4)	123.3(4)	N(2)-C(6)-C(8)	114.0(3)
N(2)-C(6)-C(7)	110.1(3)	C(8)-C(6)-C(7)	113.6(3)
N(3)-C(9)-C(11)	114.0(3)	N(3)-C(9)-C(10)	110.1(3)
C(11)-C(9)-C(10)	112.3(3)	C(13)-C(12)-P(1)	114.3(2)
C(12)-C(13)-C(13)#1	112.8(4)	C(15)-C(14)-C(19)	119.2(3)
C(15)-C(14)-P(1)	118.0(3)	C(19)-C(14)-P(1)	122.8(3)
C(16)-C(15)-C(14)	120.1(3)	C(17)-C(16)-C(15)	120.3(4)
C(16)-C(17)-C(18)	119.8(4)	C(19)-C(18)-C(17)	120.7(4)
C(18)-C(19)-C(14)	120.0(3)	C(21)-C(20)-P(1)	110.5(2)
C(23)-C(21)-C(20)	118.9(3)	C(23)-C(21)-C(22)	122.3(3)
C(20)-C(21)-C(22)	118.6(3)	O(1)-C(23)-C(21)	125.9(3)
O(1)-C(23)-O(2)	115.9(3)	C(21)-C(23)-O(2)	118.1(3)
O(3)-C(26)-C(27)	119.46(16)	O(3)-C(26)-C(31)	118.46(16)
C(27)-C(26)-C(31)	120.0	C(26)-C(27)-C(28)	120.0
C(26)-C(27)-C(32)	121.12(17)	C(28)-C(27)-C(32)	116.65(17)
C(29)-C(28)-C(27)	120.0	C(28)-C(29)-C(30)	120.0
C(28)-C(29)-C(40)	119.97(19)	C(30)-C(29)-C(40)	119.70(19)
C(31)-C(30)-C(29)	120.0	C(30)-C(31)-C(26)	120.0
C(30)-C(31)-C(36)	116.95(17)	C(26)-C(31)-C(36)	121.03(17)
C(34)-C(32)-C(35)	110.7(3)	C(34)-C(32)-C(33)	107.1(3)
C(35)-C(32)-C(33)	105.5(3)	C(34)-C(32)-C(27)	110.4(3)
C(35)-C(32)-C(27)	111.3(3)	C(33)-C(32)-C(27)	111.8(3)
C(38)-C(36)-C(39)	110.4(3)	C(38)-C(36)-C(37)	106.7(3)
C(39)-C(36)-C(37)	106.3(3)	C(38)-C(36)-C(31)	111.1(2)
C(39)-C(36)-C(31)	109.3(3)	C(37)-C(36)-C(31)	112.8(3)
O(4)-C(41)-C(46)	120.3(3)	O(4)-C(41)-C(42)	120.8(3)
C(46)-C(41)-C(42)	118.9(3)	C(43)-C(42)-C(41)	118.3(3)
C(43)-C(42)-C(47)	118.9(3)	C(41)-C(42)-C(47)	122.8(3)
C(44)-C(43)-C(42)	123.5(3)	C(43)-C(44)-C(45)	117.5(3)
C(43)-C(44)-C(55)	121.6(3)	C(45)-C(44)-C(55)	121.0(3)
C(44)-C(45)-C(46)	123.0(3)	C(45)-C(46)-C(41)	118.8(3)
C(45)-C(46)-C(51)	118.7(3)	C(41)-C(46)-C(51)	122.5(3)
C(50)-C(47)-C(42)	110.3(3)	C(50)-C(47)-C(49)	106.3(3)
C(42)-C(47)-C(49)	113.0(3)	C(50)-C(47)-C(48)	112.2(3)
C(42)-C(47)-C(48)	109.0(3)	C(49)-C(47)-C(48)	105.9(3)
C(53)-C(51)-C(54)	110.8(3)	C(53)-C(51)-C(52)	106.7(3)
C(54)-C(51)-C(52)	106.9(3)	C(53)-C(51)-C(46)	110.0(3)
C(54)-C(51)-C(46)	109.9(3)	C(52)-C(51)-C(46)	112.5(3)
C(57)-C(56)-C(61)	120.0	C(56)-C(57)-C(58)	120.0
C(57)-C(58)-C(59)	120.0	C(60)-C(59)-C(58)	120.0
C(59)-C(60)-C(61)	120.0	C(60)-C(61)-C(56)	120.0
C(67)-C(62)-C(63)	120.7(5)	C(64)-C(63)-C(62)	119.3(5)

C(65)-C(64)-C(63)	119.5(5)	C(64)-C(65)-C(66)	121.1(5)
C(67)-C(66)-C(65)	119.2(5)	C(62)-C(67)-C(66)	120.1(5)
C(69)-C(68)-C(73)	120.3(5)	C(68)-C(69)-C(70)	119.1(5)
C(71)-C(70)-C(69)	121.1(5)	C(70)-C(71)-C(72)	119.8(5)
C(71)-C(72)-C(73)	119.5(5)	C(68)-C(73)-C(72)	120.2(5)
C(1)-N(1)-P(1)	138.9(3)	C(1)-N(2)-C(3)	108.0(3)
C(1)-N(2)-C(6)	123.0(3)	C(3)-N(2)-C(6)	126.0(3)
C(1)-N(3)-C(2)	109.9(3)	C(1)-N(3)-C(9)	121.2(3)
C(2)-N(3)-C(9)	128.0(3)	C(23)-O(1)-Al(1)	131.8(2)
C(23)-O(2)-C(24)	115.2(3)	C(26)-O(3)-Al(1)	158.15(18)
C(41)-O(4)-Al(1)	164.9(2)	N(1)-P(1)-C(12)	105.23(17)
N(1)-P(1)-C(14)	113.49(17)	C(12)-P(1)-C(14)	107.07(16)
N(1)-P(1)-C(20)	116.36(16)	C(12)-P(1)-C(20)	105.61(16)
C(14)-P(1)-C(20)	108.35(17)		

#1 -x+1,-y+1,-z

Supplementary References

1. Allen, R. D., Long, T. E. & McGrath, J. E. Preparation of High Purity, Anionic Polymerization Grade Klkyl Methacrylate Monomers. *Polym. Bull.* **15**, 127–134 (1986).
2. Kuhn, N. & Kratz, T. Synthesis of Imidazol-2-ylidenes by Reduction of Imidazole-2(3*H*)-thiones**.** *Synthesis*, **6**, 561-562 (1993).
3. Tamm, M., Petrovic, D., Randoll, S., Beer, S., Bannenberg, T., Jones, P. G. & Grunenberg, J. Structural and theoretical investigation of 2-iminoimidazolines – carbene analogues of iminophosphoranes. *Org. Biomol. Chem.* **5**, 523-530 (2007).
4. Stapleto, R. A., Al-Humydi, A., Chai, J., Galan, B. R. & Collins, S. Sterically Hindered Aluminum Alkyls: Weakly Interacting Scavenging Agents of Use in Olefin Polymerization. *Organometallics* **25**, 5083–5092 (2006).
5. Faingol'd, Evgeny E., Bravaya, Natalia M., Panin, Andrei N., Babkina, Olga N., Saratovskikh, Stanislav L. & Privalov, Victor I. Isobutylaluminum aryloxides as metallocene activators in Homo- and copolymerization of olefins. *J. Appl. Polym. Sci.* **133**, 43276 (2016).
6. Long, R. J., Jones, D. J., Gibson, V. C. & White, A. J. P. Zirconium Complexes Containing Tetradentate O,P,P,O Ligands: Ethylene and Propylene Polymerization

Studies. *Organometallics* **27**, 5960-5967 (2008).

7. Bai, Y., Wang, H., He, J. & Zhang, Y. Rapid and Scalable Access to Sequence-Controlled DHDM Multiblock Copolymers by FLP Polymerization. *Angew. Chem. Int. Ed*. **59**, 11613-11619 (2020).
